# Multifunctional molecular agent for tau-targeted combinational therapy of Alzheimer’s disease

**DOI:** 10.1016/j.jbc.2025.110583

**Published:** 2025-08-12

**Authors:** Junjie Wu, Keke Chai, Ying Tu, Kang Fang, Shuo Shi, Xiaochun Hu, Jihong Liu, Tianming Yao

**Affiliations:** 1 School of Chemical Science and Engineering, Tongji University, Shanghai, PR China; 2 Department of Neurology, Shanghai Fifth People’s Hospital, Fudan University, Shanghai, PR China; 3 Ningxia Key Laboratory of Green Catalytic Materials and Technology, College of Chemistry and Chemical Engineering, Ningxia Normal University, Guyuan, PR China

**Keywords:** multifunctional molecules, tau inhibitors, copper chelators, oxidative stress, gallic acid, cyclen scaffold

## Abstract

Tau aggregation inhibitors or neurotoxic-metal chelators have been extensively studied as potential treatment for Alzheimer’s disease. However, it is a great challenge to improve their therapeutic effects while reducing neurotoxicity. Herein, we designed and synthesized two new compounds, (1,4,7,10-tetraazacyclododecane-1,4,7,10-tetrayl)tetrakis((3,4,5-trihydroxyphenyl)methanone) (4GA) and (4,10-dimethyl-1,4,7,10-tetraazacyclododecane-1,7-diyl)bis((3,4,5-trihydroxyphenyl)methanone) (2GA). Each molecule is composed of a 1,4,7,10-tetraazacyclododecane (cyclen) core as the supramolecular chelator to copper ions, attached by 2 or 4GAllic acid polyphenol arms to capture tau peptide chain like a molecular hairpin. We interestingly found that 4GA and 2GA could inhibit self-aggregation through blocking the misfolding of tau peptide, suppress the promotion of Cu^2+^ on tau aggregation by removing Cu^2+^ from dyshomeostasis, and clear reactive oxygen species triggered by Cu^2+^ using their reductive gallic acid groups. More significantly, the synthesized compounds exhibit remarkable efficiency on both *in vitro* and at cellular level. Our rational design of multifunctional therapeutic agent that simultaneously targets tau misfolding process, copper dyshomeostasis, and oxidative stress of reactive oxygen species, may hold considerable implications for the treatment of Alzheimer’s disease.

Tauopathies are a group of adult-onset neurodegenerative diseases, including Alzheimer’s disease (AD). They are defined by intracellular aggregation and transcellular propagation of protein tau (1). While tau normally plays an important role in stabilizing the microtubule network of cytoskeleton, its dissociation from microtubules and eventual aggregation into pathological deposits is an area of intense focus for therapeutic development (2). In contrast to other amyloid-forming proteins, tau aggregation is not occurring spontaneously at physiological conditions, possibly because of electrostatic repulsion of cationic amino acid residues and a rate-limiting conformational barrier (3). Misfolding and subsequent positive charge diminishing on peptide chain are likely the main cause of the aggregation into fibrillar deposits. Tau aggregation undergoes along multiple pathways. Several factors can give rise to the pathologic changes of tau protein, including hyperphosphorylation, dyshomeostasis of biometals, and oxidative stress (OS) (4). Therefore, new investigational compounds targeting each of the key factors could be exploited for the treatment of tauopathies, the misfolding disorders of tau protein.

Neurofibrillary tangles (NFTs) found within neurons in cases of AD are formed by hyperphosphorylated tau protein (5). Tau fibrillization can be induced by reducing positive charge through extensive phosphorylation (6). Therefore, attempts have been made to invent therapeutic agents by inhibiting kinase and enhancing phosphatase. However, the clinical trials demonstrated that treatments focusing solely on hyperphosphorylation of tau achieved limited success for AD therapy (7, 8). More importantly, *in vitro* experiments later exhibited that nonphosphorylated tau protein fragments could also aggregate under the induction of polyanions (9, 10, 11).

Molecular inhibitor directly blocking the process of tau aggregation provides an attractive therapeutic possibility as a disease-modifying treatment of neurodegenerative diseases. By preventing the early conformational changes, and stabilizing the soluble monomeric protein, molecular inhibitors effectively block the pathway of tau aggregation from peptide misfolding, initial oligomeric nucleation (seeds), and subsequent elongation to large amyloid fibrils. Much investigation has focused on small natural molecules, rich in aromatic groups (like polyphenols), because normal biological molecules found in food or herbal extracts usually exhibit high availability, stability, and low side effects. However, because of the weak potency, complex structural analogs, and limited synergistic functionality, only a small proportion of natural products have been applied to the treatment of diseases (12). It is of great significance to modify the structure of natural products and introduce new functional groups on the premise of preserving biological activity. Consequently, a number of synthetic tau aggregation inhibitors were prepared, and found to be effective, among which several antiaggregants underwent clinical evaluation. However, the ineffectiveness as well as adjoint toxicity because of superficial understanding of the inhibition mechanism has hindered drug development. Previously, we have investigated tannic acid and a group of glucose gallate derivatives as tau aggregation inhibitors and found they exhibit high inhibitory efficiency on tau aggregation *in vitro* (13). These star-shaped molecules have a biocompatible glucose core surrounded by several gallic acid (GA) polyphenol arms, which can bind to peptide chains at different sites, probably through hydrogen bonds and π−π stacking. Like a hairpin, these glucose gallates effectively inhibit tau aggregation through a dynamic mechanism, by elevating rate-limiting conformational barrier in tau misfolding process.

Dyshomeostasis of biometals is closely associated with the clinical development of AD symptoms, which might be a potential therapeutic target. In the brain of AD patients, the abnormally accumulated metal elements, particularly copper, have been proven to be enriched in the amyloid plaques and NFTs, suggesting a crucial role of copper in the pathogenesis of AD (14). The interaction between Cu^2+^ and tau residues stimulates tau aggregation and intracellular reactive oxygen species (ROS) production, finally inducing neuronal cell death and cognitive impairment (15). Studies have shown that erroneous deposition of copper promotes tau hyperphosphorylation, and the copper chelators attenuate tau phosphorylation in human neuroblastoma cells (5). For example, the chelator clioquinol was confirmed to disrupt the Aβ aggregation triggered by Cu^2+^ and improve cognition in mouse models (16). However, the widespread use of clioquinol has been terminated because of the adverse side effect of severe neurotoxicity (17).

OS refers to elevated levels of intracellular ROS, a pathological characteristic of tauopathy. It is a correlative process accompanied by copper dyshomeostasis for AD development. Evidence has shown that metal ions could bind to cysteine residues of tau and produce ROS, accompanied by the formation of disulfide bond (18). Accumulation of ROS can directly stimulate tau hyperphosphorylation and aggregation (19). Studies also disclosed the link between mitochondria-mediated OS and pathological aggregation/accumulation of tau oligomers in AD (20).

To the best of our knowledge, however, the design of therapeutic agents based on each of the aforementioned process has progressed slowly so far. Considering that tau aggregation undergoes along multipathways with a synergistic mechanism, a therapeutic agent targeting multiple processes logically represents a promising strategy for AD treatment (21). Multifunctional molecular agent for combinational tau-targeted therapy of AD is highly desired and remains to be explored urgently.

Herein, we combine the functional fragments, targeting respectively to tau misfolding/aggregation inhibitor, metal ion chelator, and ROS scavenger, into a single molecule. By referring our former research on tannic acid and glucose gallate derivatives, two novel macrocyclic compounds, (1,4,7,10-tetraazacyclododecane-1,4,7,10-tetrayl)tetrakis((3,4,5-trihydroxyphenyl) methanone) (4GA) and (4,10-dimethyl-1,4,7,10-tetraazacyclododecane-1,7-diyl)bis ((3,4,5-trihydroxyphenyl) methanone) (2GA), were synthesized. Each molecule is composed of a 1,4,7,10-tetraazacyclododecane (cyclen) core and two or four surrounding galloyl groups incorporated by amide bond. As illustrated in Figure 1, the tetraazacyclic structure acts as copper chelator by supramolecular recognition, whereas the galloyl group acts as a ROS scavenger through reduction process. Meanwhile, the star-shaped molecule, with several GA polyphenol arms, which can bind to tau peptide chain at different sites, acts as tau misfolding inhibitor like a “molecular hairpin.” Surprisingly, we found that the novel synthetic molecules showed higher inhibitory efficiency on tau aggregation *in vitro* and at the cell level. Our results offer a rational strategy for designing the anti-AD drugs simultaneously targeting tau aggregation, dyshomeostasis of Cu^2+^, and ROS production.

**Figure 1:**
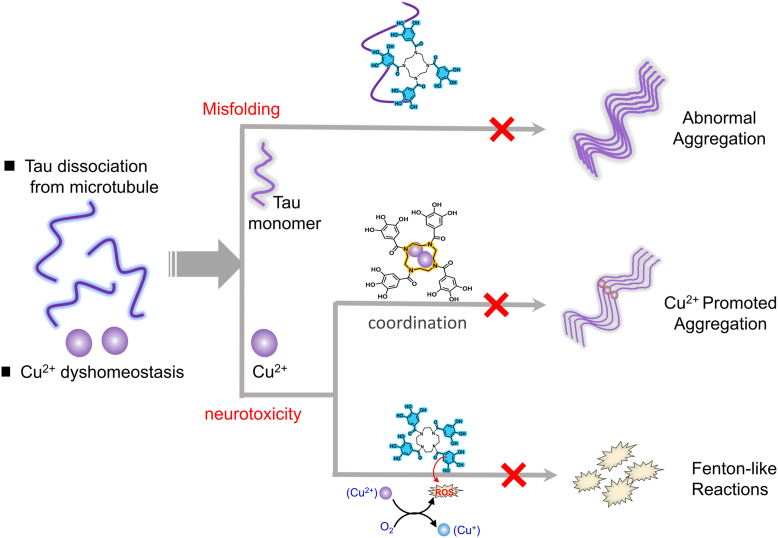
**Roadmap of the multifunctional tau-targeted molecular agent for combinational therapy of Alzheimer’s disease (AD).** Tau dissociation and metal dyshomeostasis are important factors that give rise to the pathologic changes in AD: tau misfolding/aggregation and neurotoxicity. The title compound can (i) suppress tau misfolding by catching peptide chain like a hairpin as a direct inhibitor; (ii) remove Cu^2+^ through chelation and suppress Cu^2+^ promotion on tau aggregation; (iii) clear reactive oxygen species from Cu^2+^-induced Fenton-like reaction by its reductive phenol groups, retard tau aggregation through oxidative stress mechanism.

## Results

### Synthesis and characterization of *N*-galloyl-substituted azacrown as the multifunctional tau-targeted molecular agent

**Figure 2:**
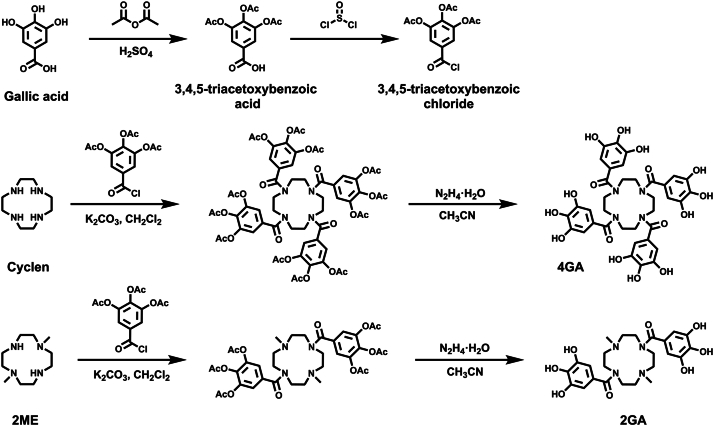
**The synthetic route of the title compounds.** (1,4,7,10-tetraazacyclododecane-1,4,7,10-tetrayl)tetrakis((3,4,5-trihydroxyphenyl) methanone) (4GA) and (4,10-dimethyl-1,4,7,10-tetraazacyclododecane-1,7-diyl)bis ((3,4,5-trihydroxyphenyl) methanone) (2GA).

The synthetic route of the title compounds is demonstrated in Figure 2. To start, the phenolic hydroxyl group on GA was protected using acetic anhydride to yield acetyl-protected GA. Then, the carboxylic acid group was chlorinated using SOCl_2_. Subsequently, 3,4,5-triacetoxybenzoic chloride reacted with cyclen, followed by deprotection using hydrazine hydrate, to give 4GA as the final product. Meanwhile, if the compound 3,4,5-triacetoxybenzoic chloride reacted with *N*-methylated cyclen (1,4,7-trimethyl-1,4,7,10-tetraazacyclododecane [2ME]), the reaction resulted in 2GA as the end product. It is notable that 4GA contains four GA groups, whereas 2GA contains only two GA groups. The new compounds, 4GA and 2GA, were characterized through ^1^H and ^13^C NMR, as well as electrospray ionization (ESI)–mass spectrometry, as described in ESI (Figs. S1–S7).

The synthetic compounds, 4GA and 2GA, are soluble in polar organic solvent such as dimethyl sulfoxide (DMSO) and slightly soluble in water. Therefore, in the following experiments, they were first dissolved in DMSO and further diluted in an excess of buffer. Moreover, UV–visible showed that both 4GA and 2GA remain stable at 72 h in Hepes (50 mM, pH 7.4, 0.2% DMSO) (Fig. S8). Logically, 1,4,7,10-tetramethyl-1,4,7,10-tetraazacyclododecane (4ME) and GA (compound structure was shown in Fig. S1) are considered as the precursor of 4GA and 2GA and therefore used as a comparative study to confirm whether the ancillary ligand had any effect on aggregation.

### The coordination properties of *N*-galloyl-substituted azacrown to Cu^2+^ and their affiliation abilities to tau peptide

As the novel multifunctional inhibitor of tau aggregation designed by us, the new compounds consist of two functional structures, the azacrown (cyclen) central core with supramolecular recognition to Cu^2+^, and the surrounding 2 or 4GAlloyl side arms, the reductive aromatic polyphenol structure, with high affinity to tau peptide chain. Prior to evaluating their biochemical effects on tau aggregation, we begin our systematic investigation from their coordination properties to Cu^2+^ and binding affinity to tau peptide.

**Figure 3:**
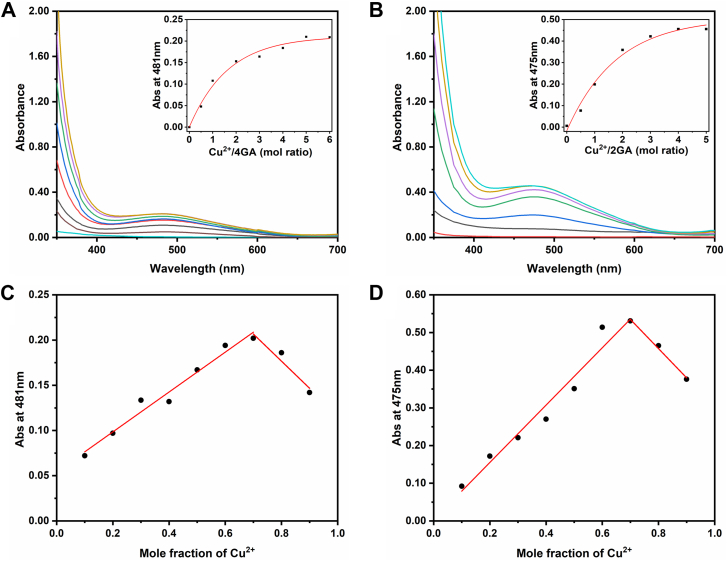
**UV spectrum and stoichiometry of complex.***A*, UV spectrum of 4GA (1 mM in DMSO) with different molar ratio of CuCl_2_ (0–6 equivalents); (*B*) UV spectrum of 2GA (1 mM in DMSO) with different molar ratios of CuCl_2_ (0–5 equivalents). *A*: absorbance at 481 nm. *B*: absorbance at 475 nm. *C*, determination of the stoichiometry of 4GA–Cu^2+^ complex (5 mM) by Job’s method. *D*, determination of the stoichiometry of 2GA–Cu^2+^ complex (5 mM) by Job’s method. DMSO, dimethyl sulfoxide; 2GA, (4,10-dimethyl-1,4,7,10-tetraazacyclododecane-1,7-diyl)bis ((3,4,5-trihydroxyphenyl) methanone); 4GA, (1,4,7,10-tetraazacyclododecane-1,4,7,10-tetrayl)tetrakis((3,4,5-trihydroxyphenyl) methanone).

The UV–visible spectrum was used to reveal their coordination properties to Cu^2+^. As shown in Figure 3, *A* and *B*, a weak broad band in the visible range at 400 to 650 nm, attributed to metal-centered d–d transitions, was observed after Cu^2+^ with various stoichiometric ratios added to the 4GA or 2GA solution (1 mM in DMSO). The increased absorption at 481 nm and 475 nm (Fig. 3, *A* and *B*, inset) definitely indicated the complexation of 4GA–Cu^2+^ and 2GA–Cu^2+^, respectively. The coordination stoichiometry of 4GA and 2GA with Cu^2+^ was determined by Job’s plot analysis (Fig. 3, *C* and *D*). For both 4GA–Cu^2+^ and 2GA–Cu^2+^, the isosbestic point appeared at 0.7 mol fraction of Cu^2+^ suggests that each macrocyclic molecule can bind with two Cu^2+^ ions. However, their macrocyclic precursors (cyclen and *N*-methylated cyclen 2ME]) were evaluated to coordinate with Cu^2+^ by 1:1 mode (Fig. S9). The different coordination mode with Cu^2+^ was supposed to be the synergistic coordination effects of galloyl side arms, in addition to the central azacrown structure.

The affinity of the synthetic compounds toward tau peptide was evaluated by endogenous fluorescence quenching experiment. The *N*-galloyl-substituted azacrown (ligand) can quench the endogenous fluorescence of tyrosine residues (306–310, VQIVY) on R3 peptide chain (receptor), if there exists a definite interaction between the ligand and the receptor. With titration of 4GA or 2GA (ligands) into the solution of tau peptide R3 (receptors), the endogenous fluorescence (at 310 nm) of tyrosine residues on R3 peptide chain was gradually quenched (data provided in ESI, Fig. S11). The data were then fitted by the Stern−Volmer formula to give the quenching constants (*K*_b_) (Fig. S12). The quenching constants (*K*_b_) and binding sites (*n*) of 4GA or 2GA to R3 peptide in the temperatures 25 °C and 37 °C are presented in Table 1.

**Table 1:** Intrinsic tau protein quenching constants of R3–4GA and R3–2GA interaction at 25 °C and 37 °C obtained from the Hill equation

T (°C)	*K*_*b*_ (10^5^ M^−1^)		*n*	
	4GA	2GA	4GA	2GA
37	50.77 ± 3.23	3.05 ± 0.18	1.54 ± 0.07	1.32 ± 0.04
25	6.01 ± 0.23	0.49 ± 0.03	1.33 ± 0.05	1.14 ± 0.05

Notably, 4GA exhibited high binding ability to tau protein (50.77 × 10^5^ M^−1^) at 37 °C, which is much higher than that of 2GA (3.05 × 10^5^ M^−1^) as well as other reported natural products, such as folic acid (2.3 × 10^5^ M^−1^) and cannabidiol (4.21 × 10^4^ M^−1^) (22, 23).These results demonstrated that with the number of phenol groups increased, the binding ability of inhibitors to tau protein became stronger, whereas GA itself showed negligible binding ability.

### Inhibitory effects of *N*-galloyl-substituted azacrown on heparin-induced R3 aggregation

**Figure 4:**
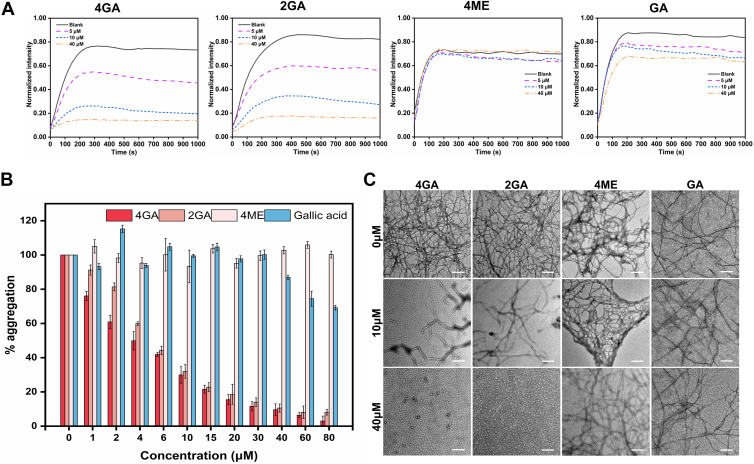
**Kinetic curves, dose−response inhibitory effects, and TEM images.***A*, kinetic curves of the R3 (15 mM) aggregation, monitored by ThS fluorescence assay with different concentrations of 4GA, 2GA, 4ME, and GA, respectively. *B*, dose−response inhibitory effects of R3 aggregation monitored by normalized ThS fluorescence intensity. The aggregation of R3 (15 mM) with different concentrations of 4GA, 2GA, GA, and 4ME was induced by heparin (3.8 mM) and incubated for 3 h at 37 °C. *C*, TEM images of R3 fibrils upon addition of 4GA, 2GA, 4ME, and GA at concentrations of 0, 10, and 40 mM. Negatively stained with uranyl acetate. The data were reported as the mean ± SD, n = 3. Scale bar represents 200 nm. GA, gallic acid; 2GA, (4,10-dimethyl-1,4,7,10-tetraazacyclododecane-1,7-diyl)bis((3,4,5-trihydroxyphenyl)methanone); 4GA, (1,4,7,10-tetraazacyclododecane-1,4,7,10-tetrayl)tetrakis((3,4,5-trihydroxyphenyl)methanone); 4ME, 1,4,7,10-tetramethyl-1,4,7,10-tetraazacyclododecane; TEM, transmission electron microscopy; ThS, thioflavin S.

The inhibition effect of 4GA–2GA on R3 aggregation was explored by thioflavin S (ThS) assay (24). In general, the kinetics of R3 aggregation in the absence or the presence of 4GA, 2GA was assessed by measuring the emission intensity of ThS at 500 nm, which reflects the amount of R3 aggregates. As the control experiments, the aggregation kinetics of 4ME and GA were also carried out. While induced by heparin, R3 aggregates rapidly at 37 °C, and the fluorescence of ThS reaches a maximum after an average time of 200 s (Fig. 4*A*). Based on the aforementioned conditions, the inhibitory ability of our synthetic compounds at three different concentrations (5, 10, and 40 μM) was tested. It was found that both 4GA and 2GA strongly inhibited R3 assembly, whereas 4ME or GA was merely able to inhibit R3 aggregation.

Next, the dose–response inhibitory effects of our synthetic compounds on R3 aggregation were investigated. The detailed experimental operation was descripted in ESI. The fluorescence spectra are demonstrated in Fig. S13. The apparent values of IC_50_ were calculated according to the dose−response curve. The IC_50s_ of 4GA and 2GA on heparin-induced R3 aggregation were determined to be 3.8 μM and 5.5 μM, respectively. Notably, the IC_50_ of both 4GA and 2GA are much higher than GA itself (more than 80 μM), close to the value of tannic acid (3.5 μM), as reported earlier (25).

To further confirm the inhibitory efficiency of 4GA and 2GA on R3 fibril formation, the morphology of heparin-induced R3 aggregates at different conditions was characterized by transmission electron microscopy (TEM) (Fig. 4*C*). After incubation, the mature fibrils of R3 were interweaved and serried in the absence of 4GA or 2GA (0 μM). In the presence of 10 μM 4GA or 2GA, smaller numbers of short R3 filaments were observed. Moreover, the formation of R3 fibrils treated by 40 μM inhibitors was strongly suppressed, and the fibrillization products looked like short fragments rather than fibrils. However, the TEM image of 4ME and GA on R3 aggregation showed that GA could hardly change the number and length of R3 fibrils even at high concentrations. Consistent with the results of ThS assay, TEM analyses also indicated that 4GA and 2GA inhibited R3 aggregation.

Logically as a control experiment, we additionally investigated the inhibitory effects of the precursor compounds, the *N*-alkylated azacrown derivatives, 1-methyl-1,4,7,10-tetraazacyclododecane (1ME), 2ME, 1,4,7-trimethyl-1,4,7,10-tetraazacyclododecane (3ME), and 4ME, on heparin-induced aggregation of R3. After introducing them into the incubation solution of R3 mixed with heparin, the ThS fluorescence intensity at 500 nm had little change, suggesting that these derivatives did not hinder the aggregation of R3 peptides (Fig. S14). This result implies that the inhibitory effect of our synthesized compound (4GA or 2GA) on tau aggregation results from the multiple GA side arms, rather than the central macrocyclic structure. Furthermore, it also confirmed the rationality of our molecular design strategy that constructs a star-shaped molecular inhibitor using azacrown as a central scaffold plus peripheral side arms for catching tau peptide chains.

### *N*-substituted azacrown derivatives suppress the promotion of Cu^2+^ on R3 aggregation

**Figure 5:**
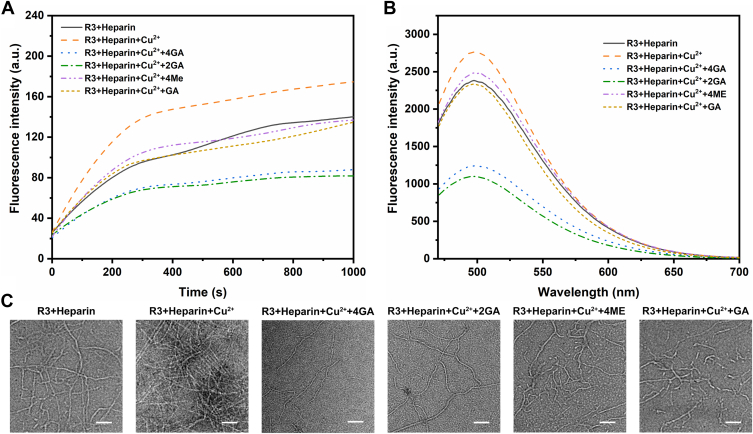
**Kinetic curves and TEM image of R3 aggregation with 4GA or 2GA.***A*, kinetic curves of the R3 (15 μM) aggregation monitored by ThS fluorescence assay in different conditions. *B*, ThS fluorescence intensity of R3 (15 μM) incubated for 3 h in different conditions. *C*, TEM image of R3 fibrils in different conditions. Negatively stained with uranyl acetate. Scale bar represents 200 nm. Final concentrations of R3, Cu^2+^, and inhibitors are 15, 3.8, and 10 μM, respectively. All aggregations were initiated by heparin (3.8 μM). 2GA, (4,10-dimethyl-1,4,7,10-tetraazacyclododecane-1,7-diyl)bis((3,4,5-trihydroxyphenyl)methanone); 4GA, (1,4,7,10-tetraazacyclododecane-1,4,7,10-tetrayl)tetrakis((3,4,5-trihydroxyphenyl)methanone); TEM, transmission electron microscopy; ThS, thioflavin S.

Metal ions, especially Cu^2+^, are believed to be highly compatible with cysteine residues of tau peptide as a coordination center (26). Accompanied by the formation of intermolecular S–Cu–S bridging coordination bond, or intermolecular disulfide bond, these metal ions have been proven to promote tau aggregation (27). After confirming that 4GA and 2GA can effectively remove copper ions through coordination, we further explore their effects on R3 aggregation under the promotion of Cu^2+^. As shown in Figure 5, *A* and *B*, the kinetics of R3 aggregation were monitored by the time-dependent ThS fluorescence, and the general inhibition effects on R3 aggregation in solution incubation were examined by the ThS fluorescence intensity at 500 nm. Heparin was used to initiate the aggregation (15). In the presence of Cu^2+^, a higher ThS fluorescence intensity was observed compared with the absence of Cu^2+^, indicating that Cu^2+^ significantly promoted the aggregation of R3. Interestingly, when 4GA or 2GA was introduced to the R3 aggregation solution in the presence of Cu^2+^, the ThS fluorescence intensity dropped dramatically, to a level lower than the case of R3 aggregation in the absence of Cu^2+^. As a comparison, the effects of the precursor compounds: 4ME, 3ME, 2ME, and 1ME, on Cu^2+^-promoted R3 aggregation were also examined (Fig. S15). When these *N*-alkylated azacrown compounds were introduced to the R3 aggregation solution in the presence of Cu^2+^, the ThS fluorescence intensity also decreased, but the decrease was much smaller compared with the case of 4GA or 2GA, only to a level close to the case of R3 aggregation in the absence of Cu^2+^. This result is predictable, because *N*-galloyl-substituted azacrown, 4GA or 2GA, has double effects: (i) suppressing tau aggregation by its galloyl arms and (ii) eliminating Cu^2+^ promotion on tau aggregation by its macrocyclic structure. On the other hand, *N*-alkylated azacrown, 4ME and others can only suppress Cu^2+^ promotion on tau aggregation by its macrocyclic structure but cannot suppress tau aggregation because its alkyl substituted arms do not have the ability to bind peptide chains.

Subsequently, the inhibition effects of 4GA–2GA, as well as their precursor compounds (4ME and GA), on Cu^2+^-promoted R3 aggregation were further morphologically confirmed by TEM. As shown in Figure 5*C*, in the absence of Cu^2+^, R3 aggregation was induced by heparin to give long and compact fibrils. In the presence of Cu^2+^, R3 accumulated vigorously under the induction of heparin, to give crowded, long, and twisted filaments, indicating that Cu^2+^ could stimulate the fibrillization of R3. With the addition of 4GA or 2GA into the R3/Cu^2+^ aggregation solutions, the resulting filaments become sparse and small. However, the influence of 4ME or GA on Cu^2+^-promoted R3 aggregation was small, because these compounds could only suppress Cu^2+^ promotion effect; their effect on R3 self-aggregation was almost negligible.

### Cytotoxicity of 4GA and 2GA on human neuroblastoma cells (SK-N-SH)

**Figure 6:**
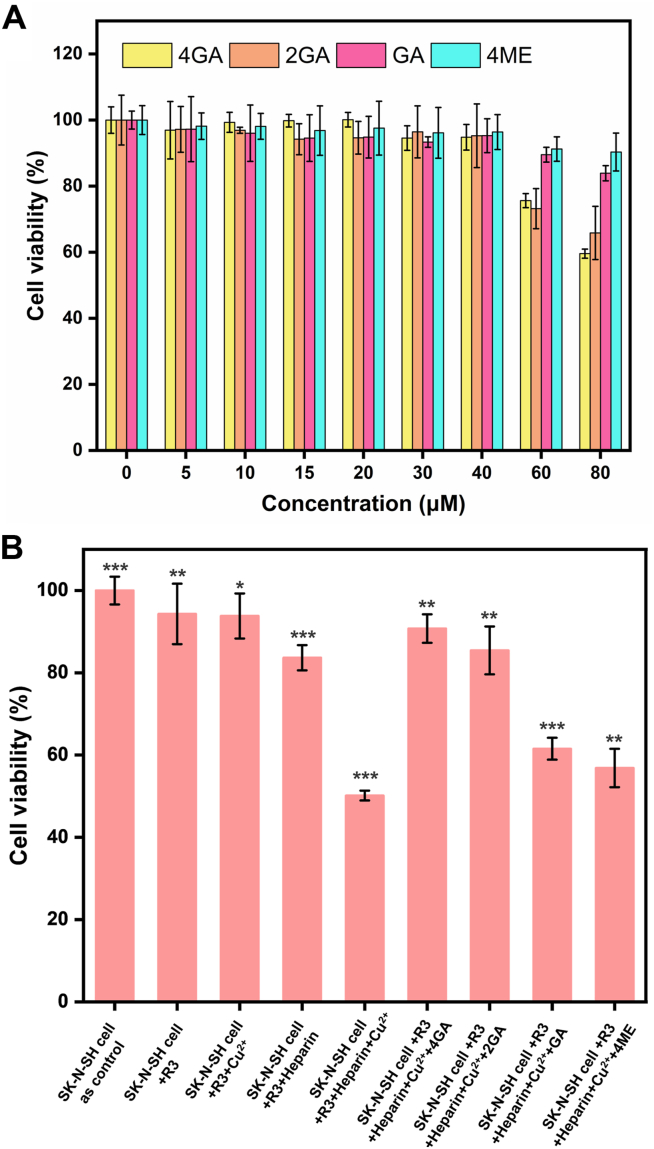
**Dose-dependent cell viability tests.***A*, dose-dependent cell viability tests of SK-N-SH cells treated with 4GA, 2GA, GA, and 4ME. *B*, cell viability of SK-N-SH cells treated with R3, R3 aggregates (R3 + heparin), Cu^2+^, 4GA, 2GA, GA, and 4ME, prepared in indicated conditions. The data were reported as the mean ± SD, n = 3. Statistical analysis was performed using a one-way ANOVA test, ∗*p* < 0.05, ∗∗*p* < 0.01, and ∗∗∗*p* < 0.001 (n = 3). GA, gallic acid; 2GA, (4,10-dimethyl-1,4,7,10-tetraazacyclododecane-1,7-diyl)bis((3,4,5-trihydroxyphenyl)methanone); 4GA, (1,4,7,10-tetraazacyclododecane-1,4,7,10-tetrayl)tetrakis((3,4,5-trihydroxyphenyl)methanone); 4ME, 1,4,7,10-tetramethyl-1,4,7,10-tetraazacyclododecane.

In general, the aforementioned *in vitro* results signified the initial success of our design strategy for constructing multifunctional inhibitors on tau aggregation. However, we still face challenges to improve their therapeutic effects while reducing neurotoxicity. Therefore, a systematic investigation on the cytotoxicity of our new synthetic compounds was carried out. Herein, human neuroblastoma cells (SK-N-SH) were utilized for standard cell viability assay (Cell Counting Kit-8 [CCK-8] assay). Figure 6*A* shows the dose-dependent cell viability of SK-N-SH cells treated with 4GA, 2GA, GA, and 4ME. It is observed that both 4GA and 2GA exhibit insignificant cytotoxicity in SK-N-SH cells, even at concentration up to 40 μM, indicating good biocompatibility. At a concentration as high as 80 μM, their cytotoxicity became remarkable, 40.5% and 34.2% of SK-N-SH cells was dead after 24 h incubation with 4GA and 2GA, respectively. As the control, the survival rate of SK-N-SH cells treated with GA or 4ME was also examined, and the results showed that their cytotoxicity was also negligible.

Literature reports (18, 19), as well as our previous study, have revealed that Cu^2+^ is a neurotoxic transition metal because it can induce Fenton-like reactions to produce ROS and promote abnormal tau aggregation to form NFTs. Meanwhile, we had proven that tau self-aggregation in SK-N-SH cells could be directly induced by the R3 aggregate (R3 + heparin) (28), which provided a more straightforward cell model for tauopathy than using other inducers (*e.g.*, VQIVYK). In this regard, we next evaluated the detoxification effect of 4GA and 2GA in the SK-N-SH cells treated with different components with possible cytotoxicity, including R3, R3 aggregates (R3 + heparin), Cu^2+^, 4GA, 2GA, GA, and 4ME in indicated conditions, as shown in Figure 6*B*. Significantly, the addition of either 4GA or 2GA to the SK-N-SH cells along with various cytotoxic components dramatically elevates the cell viability, indicating that both the compounds can attenuate neurotoxicity and protect cells against R3 aggregates or Cu^2+^. In comparison, GA and 4ME showed only a limited rescue ability.

### The scavenge ability of 4GA and 2GA on ROS

OS is one of the pathogenesis of AD caused by ROS imbalance. Evidence has shown that Cu^2+^ could bind to cysteine residues of tau and generate ROS through Fenton-like reaction, accompanied by the oxidation of thiol groups of cysteine residues, to give disulfide bond, eventually accelerating tau aggregation (18). Notably, several studies pointed out that GA could reduce nerve damage and cerebral amyloid neuropathy in the AD context (29). For these reasons, we were interested in the scavenge ability of 4GA and 2GA on ROS.

**Figure 7:**
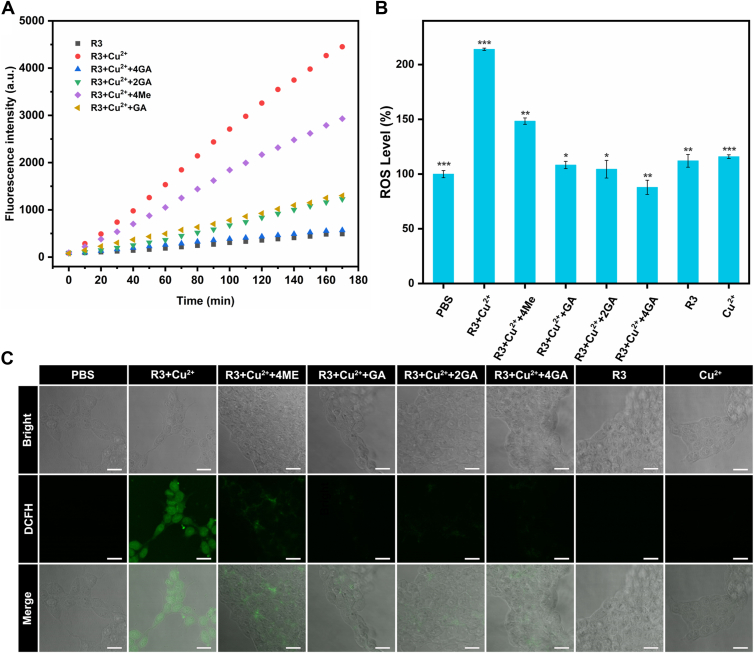
**The scavenge ability of 4GA and 2GA on ROS.***A*, time-dependent experiment for monitoring ROS level in solution at different conditions, using the fluorescent probe 2′,7′-dichlorodihydrofluorescein (DCFH). *B*, ROS levels in SK-N-SH cells treated with different conditions detected by the fluorescent probe of DCFH in flow cytometry. The data were reported as the mean ± SD, n = 3. Statistical analysis was performed using a one-way ANOVA test, ∗*p* < 0.05, ∗∗*p* < 0.01, and ∗∗∗*p* < 0.001 (n = 3). *C*, confocal laser scanning microscopy (CLSM) analysis of the intracellular ROS level stained with DCFH. Scale bar represents 12.5 μm. 2GA, (4,10-dimethyl-1,4,7,10-tetraazacyclododecane-1,7-diyl)bis((3,4,5-trihydroxyphenyl)methanone); 4GA, (1,4,7,10-tetraazacyclododecane-1,4,7,10-tetrayl)tetrakis((3,4,5-trihydroxyphenyl)methanone); ROS, reactive oxygen species.

In this experiment, the fluorescent probe, 2′,7′-dichlorodihydrofluorescein (DCFH), was used to detect the formation of ROS in solution. In Figure 7*A*, the fluorescence intensity of DCFH was drastically increased after Cu^2+^ was added to the solution of R3, demonstrating the generation of ROS. After introducing 4GA or 2GA into the solution, the DCFH fluorescence enhancement disappeared, and the fluorescence intensity significantly decreased, returning to the level observed in the R3 solution without Cu^2+^. These results indicated that 4GA or 2GA could scavenge ROS generated by Cu^2+^ involved in Fenton reaction, because of the reductive properties of GA polyphenol arms. It is worth noting that GA could also quench the DCFH fluorescence, whereas 4ME could not, indicating that reductive GA polyphenol arms play an important role in scavenging ROS.

The flow cytometry and confocal laser scanning microscopy (CLSM) were used to show the intracellular ROS production. As shown in Figure 7*B*, R3–Cu^2+^ complex boosted the ROS production in SK-N-SH cells by 2.14 ± 0.01 fold, whereas R3 or Cu^2+^ alone resulted in no significant increase. Treated with 4GA, 2GA, or GA, the ROS in SK-N-SH cells generated by R3–Cu^2+^ complex appreciably decreased, almost to the control level. On the contrary, treated with 4ME, the ROS in SK-N-SH cells generated by R3–Cu^2+^ complex did not decrease. In Figure 7*C*, CLSM displayed a conspicuous green fluorescence in SK-N-SH cells containing R3–Cu^2+^ complex, indicating a high level of intracellular ROS. Treated with 4GA, 2GA, or GA, CLSM green fluorescence in SK-N-SH cells almost disappeared. Again, the CLSM demonstrated that 4GA and 2GA showed excellent scavenging capacity against ROS. Consequently, the reductive phenolic hydroxyl groups might be a key factor for scavenging ROS triggered by R3–Cu^2+^ complex.

### The inhibitory effects of 4GA and 2GA on tau aggregation in human neuroblastoma cells (SK-N-SH)

**Figure 8:**
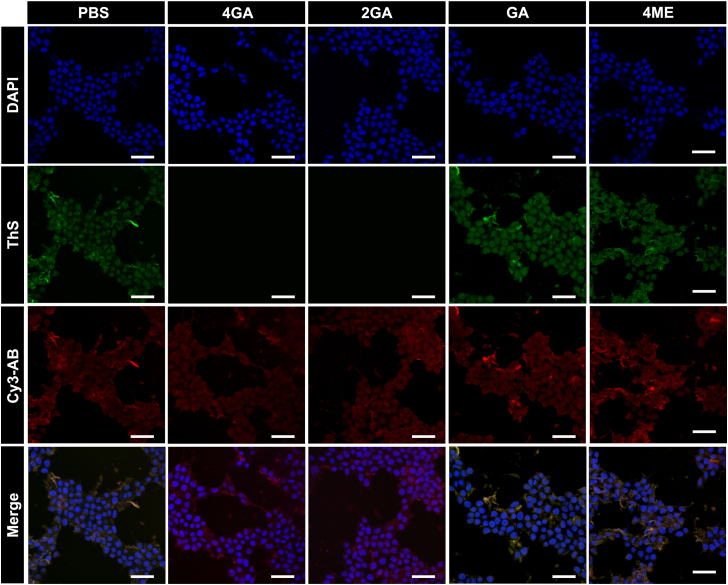
**Confocal laser scanning microscopy images of SK-N-SH cells incubated with R3 aggregates and different inhibitors.** 2-(4-Amidinophenyl)-6-indolecarbamidine dihydrochloride (DAPI) for nuclei staining (*blue*); Thioflavin S (ThS) for aggregated Tau protein (*green*); Cy3 marked anti-Tau antibody (Cy3-AB) for Pan Tau protein (all conformations of Tau) (*red*). Concentrations of R3 aggregates and inhibitors are 1.5 μM and 10 μM, respectively. Scale bar represents 25 μm.

With aforementioned systematic investigation on cytotoxicity, we finally found that 4GA and 2GA had a definite detoxification effect in the SK-N-SH cells treated with different cytotoxic components. These results prompt us to further confirm the biological functions of our synthetic compounds to inhibit intracellular tau aggregation in SK-N-SH cells, using immunofluorescence staining images. In Figure 8, the CLSM is displayed. The green fluorescence of ThS staining represented the distribution of tau fibrils formed in the tau-infected SK-N-SH cells, indicating that R3 aggregates (R3 incubated with heparin) could induce intracellular tau aggregation. The red fluorescence of antitau antibody (Cy3-AB) staining marked for Pan Tau protein. After tau-infected SK-N-SH cells were incubated with 4GA or 2GA, the green fluorescence disappeared, demonstrating that most of the intracellular tau fibrillary aggregation had been inhibited and possibly converted to other tau forms, as indicated by the red fluorescence of Cy3-AB. Comparatively, after incubation with GA and 4ME, the green fluorescence was still visible, indicating a slight inhibitory efficiency on intracellular tau aggregation in SK-N-SH cells, which is consistent with the previous studies. Finally, the fluorescence intensity was quantitatively analyzed *via* ImageJ (National Institutes of Health). As shown in Fig. S16, after being treated with 4GA and 2GA, the fluorescence intensity of ThS was reduced to only 3.3% and 4.6% against the control group, respectively. These results suggested that both 4GA and 2GA could effectively inhibit the aggregation of tau in living cells.

## Discussion

The tau fragment R3 (306VQIVYK PVDLSK VTSKCG SLGNIH HKPGGG Q336) is the core sequence and nucleation site to form tau aggregates (30). R3 has high stability and rapid aggregation potential compared with the full-length tau protein. Series of our previous works have proved that R3 aggregation behavior is fully competent to simulate full-length tau (13, 25, 28). Moreover, the presence of the two adjoining histidines makes the binding of copper to R3 stronger and the oxidative reaction activity higher than other three repeat units (R1, R2, and R4) (26). Therefore, the R3 peptide provides a convenient and reliable model for the exploration of tau aggregation inhibition.

Under pathological conditions, tau aggregation proceeds along multiple pathways and has a synergistic mechanism. On the one hand, tau fibrosis is driven by the misfolding of the peptide chain as well as the diminishing of positive charge on the peptide chain because of hyperphosphorylation. On the other hand, tau aggregation is stimulated by the abnormally accumulated Cu^2+^ in neuron through the interaction between Cu^2+^ and tau residues as well as the subsequent generation of intracellular ROS through Fenton-like reaction. Therefore, for the first time, we designed and synthesized two novel compounds; each molecule is composed of a cyclen core and 2 or 4GAllic acid polyphenol groups as the arms, named 2GA and 4GA.

Our design idea on the special molecular structure came from the following concepts: (1) Relying on a typical supramolecular recognition between azacrown and transition metal ion, cyclen has been widely used as the macrocyclic ligand toward Cu^2+^ with high thermodynamic stability (log *K*_Cu-cyclen_ = 24.8) and kinetic inertness (31). It is noteworthy that there has been report regarding cyclen as a multifunctional framework targeting the dyshomeostasis of Cu^2+^ for the treatment of AD (32). (2) We introduced multiple GA side arms into the designed compounds, because these surrounding phenolate groups can bind with peptide chains at different sites, probably through hydrogen bonds and π−π stacking, similar to the case in tannic acid or glucose gallate we reported earlier (13). Taking advantage of their novel structure, the star-shaped molecule is likely to prevent misfolding of tau peptide within self-aggregation process like a molecular hairpin. (3) With a reductive aromatic polyphenol structure, galloyl arms may also have the ability to clear up ROS triggered by tau–Cu^2+^ complex (29).

In summary, we successfully constructed two multifunctional synthetic inhibitors based on GA arms with azacrown metal–binding core, which can simultaneously target tau and Cu^2+^ for the therapy of AD. It was found that both 4GA and 2GA exhibited significant interaction with tau, which may block tau–tau interaction through preventing the conformational transition of proteins, thereby inhibiting the filament formation of R3. The results showed that the increase of GA arms could enhance the binding ability of inhibitors to tau protein. Notably, these synthetic molecules could efficiently chelate the excessive Cu^2+^, suppress the Cu^2+^-triggered tau aggregation, and reduce the intracellular ROS production. Moreover, cellular experiments demonstrated that this series of compounds performed good biocompatibility and rescue ability to restore cell viability caused by tau–Cu^2+^ species. Overall, we demonstrated that combining the tau-target pharmacophore moiety with metal chelator could be a new strategy for the development of novel multifunctional drugs for the treatment of AD.

## Experimental procedures

### Chemicals

Cyclen, GA, and SOCl_2_ were obtained from Aladdin. Acetic anhydride, hydrochloric acid, concentrated H_2_SO_4_, and hydrazine monohydrate were obtained from Sinopharm. 1ME, 2ME, 3ME, 4ME, and 3,4,5-triacetoxybenzoic acid were synthesized. R3 (^306^VQIVYK PVDLSK VTSKCG SLGNIH HKPGGG Q^336^) and rabbit anti-tau antibody were purchased from MuJin. Heparin sodium salt and ThS were obtained from Sigma–Aldrich. CCK-8 and DCFH diacetate were obtained from Beyotime. Paraformaldehyde (4%), Cy3-conjugated donkey anti-rabbit IgG, goat serum, and 0.1% Triton X-100 were purchased from BBI. And all the other reagents were purchased from Titan.

### UV spectrum and metal chelation

The chelating studies were performed on Hitachi U-2900 UV–visible spectrophotometer with a 10-mm quartz cell. For titration experiment, the UV absorption of test compound (1 mM in DMSO) was recorded in the presence of CuCl_2_ (0–6 equivalents of Cu^2+^ for 4GA, 0–5 equivalents of Cu^2+^ for 2GA, respectively). For Job’s method, a series of solution was prepared in which the total concentration of compound and Cu^2+^ was fixed (5 mM in DMSO), but their proportions of ether component varied between 0% and 100%, and the UV spectra were analyzed to determine the ratio of ligand–Cu^2+^.

### Fluorescence quenching assay

The structural changes of R3 protein upon binding to 4GA/2GA/GA was documented by fluorescence spectroscopy. Briefly, various concentrations of 4GA/2GA/GA (0, 5, 10, 15, 20, 30, 40, 60, or 80 μM) were added to the 15 μM R3 protein in 50 mM Tris–HCl buffer (pH 7.4) in a 10-mm quartz cuvette. The measurement was carried out by Hitachi F-7000 equipped with a circulating water bath at 25 and 37 °C. The fluorescence spectra of R3 protein were documented from 290 nm to 380 nm with 275 nm excitation. The widths of excitation and emission slit were set at 5 and 10 nm, respectively.

The Stern–Volmer (Formula 4.1) was used to determine the quenching mechanism, either static or dynamic quenching:

(4.1) $$\begin{array}{c} \frac{F_{0}}{F}=1+K_{SV}\left[Q\right] \end{array}$$

where *F*_0_ and *F* are the fluorescence intensity without and with quencher, [*Q*] means the concentration of quencher, and *K*_sv_ is the Stern–Volmer constant.

Furthermore, the binding constant (*K*_b_) and number of the binding sites (*n*) of quencher on R3 protein could be calculated by Hill (Equation 4.2):

(4.2) $$\begin{array}{c} \log\left[\frac{F_{0}-F}{F}\right]=\log\;K_{b}+n\;\log\;\left[Q\right] \end{array}$$

### ThS assay

#### Kinetics of R3 aggregation and inhibition

Tau peptide R3 was adjusted to a concentration of 15 μM using 50 mM Tris−HCl buffer (pH 7.4), ThS (final concentration of 10 μM), and different amounts of 4GA, 2GA, 4ME, and GA (final concentration of 0, 5, 10, and 40 μM) as the aggregation inhibitor were added in1to the reaction mixture. Aggregation was induced by adding heparin to the solution (final concentration of 3.8 μM); the kinetics of each sample was immediately monitored by a fluorescence spectrophotometer with a 10-mm quartz cell at 440/500 nm (excitation/emission). A circulating water bath was necessary to maintain the temperature at 37 °C.

#### Dose−response inhibitory effects of R3 aggregation

Tau peptide R3 was adjusted to a concentration of 15 μM using 50 mM Tris–HCl buffer (pH 7.4). Different amounts of 4GA, 2GA, 4ME, and GA (final concentration of 0, 1, 2, 4, 6, 10, 15, 20, 30, 40, 60, or 80 μM) as the aggregation inhibitors were added into the reaction mixture. Aggregation was induced by adding heparin to the solution (final concentration of 3.8 μM), After 3 h incubation at 37°C, ThS (final concentration of 10 μM) was added, and the fluorescence intensity at 440/500 nm (excitation/emission) was recorded on Hitachi F-7000 with a 10-mm quartz cell. The dose−response inhibitory effects of R3 aggregation were expressed by the aggregation percentage, which was calculated according to the following (Formula 4.3):

(4.3) $$\begin{array}{c} \text{aggregation}\;\%=\frac{F\;-{\;F}_{0}}{F_{t}\;-{\;F}_{0}}\times 100 \end{array}$$

where *F*_0_ = fluorescence intensity of R3 monomer, *F*_t_ = fluorescence intensity of R3 aggregate, and *F* = fluorescence intensity of R3 aggregate in the presence of inhibitors. The IC_50_ values were also calculated according to the dose−response inhibitory effects.

The promotion of Cu^2+^ on R3 aggregation was also inspected, similar to the aforementioned operation, unless that 3.8 μM Cu^2+^ was also included, and using 50 mM Hepes (pH 7.4) as the buffer solution.

### Transmission electron microscopy

For detecting the inhibitory efficiency of inhibitors, 15 μM R3, 3.8 μM heparin, and 4GA/2GA/4ME/GA (0, 10, and 40 μM) in 50 mM Tris–HCl buffer (pH 7.4) was incubated at 37 °C for 3 h, respectively. For exploring the effect of Cu^2+^ on R3 aggregation, 15 μM R3, 3.8 μM heparin was mixed in the presence or the absence of 3.8 μM Cu^2+^ in 50 mM Hepes buffer (pH 7.4). Then, 10 μM 4GA/2GA/4ME/GA was added to the solution. After 3 h incubation, 10 μl of each solution was applied on a carbon support membrane, followed by 10 μl of 2% uranyl acetate, and dried at room temperature. The operation was carried out on transmission electron microscope (JEM-2100, provieded by JEOL).

### Cell culture

The human neuroblastoma cell (SK-N-SH) was obtained from the Chinese Academy of Sciences stem cell bank. Cells were maintained in Dulbecco’s modified Eagle’s medium (DMEM) supplemented with 10% fetal bovine serum (FBS), 100 U/ml penicillin G, and 100 μg/ml streptomycin at 37 °C in a humidified atmosphere containing 5% CO_2_.

### Cell viability assay

Cells were plated in 96-well plate at a density of 1 × 10^4^ in 100 μl medium per well for 24 h. Then, the FBS-free medium containing the tested compounds at different concentrations was used to replace the old medium. After 24 h incubation, 100 μl of FBS-free DMEM containing 10% CCK-8 was added into each well to replace the old medium. After 2 h incubation, the absorbance at 450 nm was measured by SpectraMax iD3 (Molecular Devices).

For cell rescue assay, 30 μM R3, 7.5 μM heparin was incubated in FBS–DMEM containing 7.5 μM Cu^2+^ and different compounds (20 μM) at 37 °C for 1 h. Then, 500 μl of preincubated R3 solution was diluted with 500 μl fresh FBS–DMEM, and the mixture was used to replace the old medium after the cell attached to the 96-well plate. After cultured for 24 h, CCK-8 was used to measure the cell viability.

### Immunofluorescence staining

Tau-infected pathological model cell lines were constructed by human neuroblastoma SK-N-SH cells using R3 aggregates as inducer, referring our earlier article (28). R3 aggregates were obtained from coincubation of 15 μM R3 peptide with 3.8 μM heparin in PBS at 37 °C for 4 h. Then, the solution was diluted to 1.5 μM in the culture medium. SK-N-SH cells were seeded onto 24-well plates (1 × 10^5^/well) for 24 h. Then, 10 μM of different compounds (inhibitors) per well was added and incubated for 2 h. After changing the culture medium, these cells were treated with 1.5 μM R3 aggregates for 12 h and then further continued incubation for 12 h.

The samples were successively treated with 4% paraformaldehyde and 0.1% Triton X-100 for 15 min each and washed three times with PBS. Blocked with 5% goat serum for 30 min, the samples were incubated with rabbit anti-Tau antibody (1:200 dilution) at 4 °C for 24 h and washed three times with PBS. Then they were incubated with Cy3-conjugated Donkey Anti-Rabbit IgG (1:1000 dilution) at 37 °C for 1 h and washed three times with PBS. Subsequently, 60 μM ThS was added to stain tau aggregates for 15 min and washed three times with PBS. Finally, cells were stained with 2-(4-amidinophenyl)-6-indolecarbamidine dihydrochloride (0.02%) for 5 min to staining nuclei and washed three times with PBS. The antifade mounting medium was used for cell slides to ensure fluorescence. The fluorescence signals were monitored using a CLSM (Leica TCS SP8).

### Intracellular ROS measurement

SK-N-SH cells were seeded on a 12-well plate at a density of 1 × 10^5^/well and incubated at 37 °C for 24 h, followed by treating with 15 μM R3 in the presence or the absence of 3.8 μM Cu^2+^ in FBS-free DMEM. Two hours later, 10 μM 4GA/2GA/4ME/GA was added to the medium, respectively. After 24 h, the culture medium was removed, and 10 μM DCFH diacetate in FBS-free DMEM was added and incubated for 45 min at 37 °C. Samples were washed three times with PBS, and 4% polyformaldehyde was added and fixed at room temperature for 20 min. Cells were observed under a CLSM. For quantitative analysis, cells were harvested and washed three times with ice-cold PBS and resuspended in PBS. The fluorescence intensity of DCFH was analyzed by flow cytometry (CytoFLEX LX).

### Statistical analysis

The data were presented as the mean ± standard deviation. One-way single factorial ANOVA was performed to determine the statistical significance of the data. The statistical significance of the differences was expressed as ∗*p* < 0.05, ∗∗*p* < 0.01, and ∗∗∗*p* < 0.001.

## Data availability

All relevant data are contained within the article. In cases when mean values or representative experiments are reported, data for all individual experiments can be provided by the corresponding author upon request.

## Supporting information

This article contains supporting information.

## Conflict of interest

The authors declare that they have no conflicts of interest with the contents of this article.
