# Supplementary material for: Multifunctional molecular agent for tau-targeted combinational therapy of Alzheimer’s disease
Source: J Biol Chem. 2025 Aug 12;301(9):110583. doi: 10.1016/j.jbc.2025.110583 (PMC12455130; doi:10.1016/j.jbc.2025.110583)
Supplement: Supporting information [file mmc1.docx]

**Supporting Information**

**Multifunctional** **molecular agent for tau-targeted combinational therapy of Alzheimer’s disease**

Junjie Wu^1^, Keke Chai^1^, Ying Tu^1^, Kang Fang^1^, Shuo Shi^1^, Xiaochun Hu^1^, Jihong Liu^2*^ and Tianming Yao^1,3*^

*Correspondence: [tmyao@tongji.edu.cn](mailto:tmyao@tongji.edu.cn)；[liujihong@5thhospital.com](mailto:liujihong@5thhospital.com).

Affiliations:

1. School of Chemical Science and Engineering, Tongji University, Shanghai 200092, P.R.China;
2. Department of Neurology, Shanghai Fifth People’s Hospital, Fudan University, Shanghai, 200240, P.R.China;
3. College of Chemistry and Chemical Engineering, Ningxia Normal University, Guyuan 756099, P.R.China.

content

1. [Synthesis, characterization of new compounds by NMR and ESI-MS S2](#_Toc127985841)

2. [Spectrastabilities of the new molecules S3](#_Toc127985842)

3. Job’s plot analysis for determination of stoichiometry of macrocyclic-Cu2+ complexes  [S](#_Toc127985843)6

4. Intrinsic Fluorescence quenching experiments for determination of tau protein quenching constants  [S](#_Toc127985843)8

5. The dose−response inhibitory effects of different N-substituted azacrown on R3 aggregation investigated by ThS fluorescence (For calculation of IC50)[. S11](#_Toc127985843)

6. Inhibitory effects of N-substituted Azacrown on heparin-induced R3 aggregation, exermined by ThS fluorescence [. S12](#_Toc127985843)

7. [N-substituted azacrown suppress the promotion of Cu2+ on heparin-induced R3 aggregation S13](#_Toc127985843)

8. [Quantitatively analysis of the fluorescence intensity in the Confocal laser scanning microscopy images (Figure 7) of SK-N-SH cells incubated with R3, heparin and different inhibitors by ImageJ software S13](#_Toc127985843)

9. [References S14](#_Toc127985843)

1.Synthesis, characterization of new compounds by NMR and ESI-MS spectra

*Synthesis of (1,4,7,10-tetraazacyclododecane-1,4,7,10-tetrayl)tetrakis ((3,4,5-trihydroxyphenyl)methanone) (4GA)*

The synthesis routes of **4GA** and **2GA**, and the characterization data were given in Electronic Supporting Information (Figure S1-7)

The synthesis of **4GA** was carried out in three steps:

**Step 1.** 3,4,5-triacetoxybenzoic acid was synthesized according to the previous literature.^39^ Briefly, gallic acid (5 g, 29.4 mmol), acetic anhydride (17 mL) and conc. H_2_SO_4_ (2 drops) was heated to reflux for 1.5 h. Then, water was added to continue stirring for 3 h, follow by filtrating and drying to yield product. A mixture of 3,4,5-triacetoxybenzoic acid (2.086 g, 7 mmol) and SOCl_2_ (5.25 mL, 70 mmol) was heated at reflux for 3 h under N_2_ atmosphere. Excess of SOCl_2_ was evaporated on a rotary evaporator, and 3,4,5-triacetoxybenzoic chloride was obtained as a pale yellow solid.

**Step 2.** A mixture of cyclen (0.2 g, 1.16 mmol) and K_2_CO_3_ (1.06 g, 10 mmol) in dry CH_2_Cl_2_ (15 mL) was stirred at 0 ℃ for 0.5 h. Then, excess 3,4,5-triacetoxybenzoic chloride (all solid obtained in step 1) in dry CH_2_Cl_2_ (10 mL) was added dropwise to the solution at 0 ℃. The reaction solution was further stirred overnight at room temperature under N_2_ atmosphere. The mixture was washed with distilled water (3 × 20 mL), and the organic layer was collected, dried with anhydrous Na_2_SO_4_, and evaporated on a rotary evaporator to give the crude product.

**Step 3.** The crude product was dissolved in acetonitrile (15 ml), and then 80% hydrazine monohydrate (10 drops) was added. The reaction mixture was stirred at room temperature for 30 min, and a viscous oil product was formed. After removing the solution, distilled water (15 mL) was added to give white suspension, and 10% HCl was added dropwise to adjust the pH to 5~6. The suspension was filtered, washed with water (2 × 10 mL), methanol (2 × 10 mL) and dried under vacuum to give compound as white solid. Yield: 0.824 g, 91%. ^1^H NMR (400 MHz, DMSO-*d*_6_) δ 9.05 (s, 8H), 8.45 (s, 4H), 6.25 (s, 8H), 3.65 (m, 16H). ^13^C NMR (400 MHz, DMSO-*d*_6_) δ 171.52, 145.86, 105.79, 40.42. HRMS (ESI) Calcd. for C_36_H_36_N_4_O_16_ [M+Na]+: 803.20185; Found: 803.20180.

*Synthesis of (4,10-dimethyl-1,4,7,10-tetraazacyclododecane-1,7-diyl)bis ((3,4,5-trihydroxyphenyl)methanone) (2GA)*

The synthesis procedure of **2GA** is similar to **4GA**. The desired compound was obtained as white solid. Yield: 0.413 g, 82%. ^1^H NMR (400 MHz, DMSO-*d*_6_) δ 8.94 (br, 6H), 6.30 (s, 4H), 3.45 (s, 8H), 2.55 (s, 8H), 2.11 (s, 3H). ^13^C NMR (400 MHz, DMSO-*d*_6_) δ 171.32, 145.64, 134.08, 127.17, 106.17, 48.64. HRMS (ESI) Calcd. for C_24_H_32_N_4_O_8_ [M+H]+: 505.22929; Found: 505.22976.


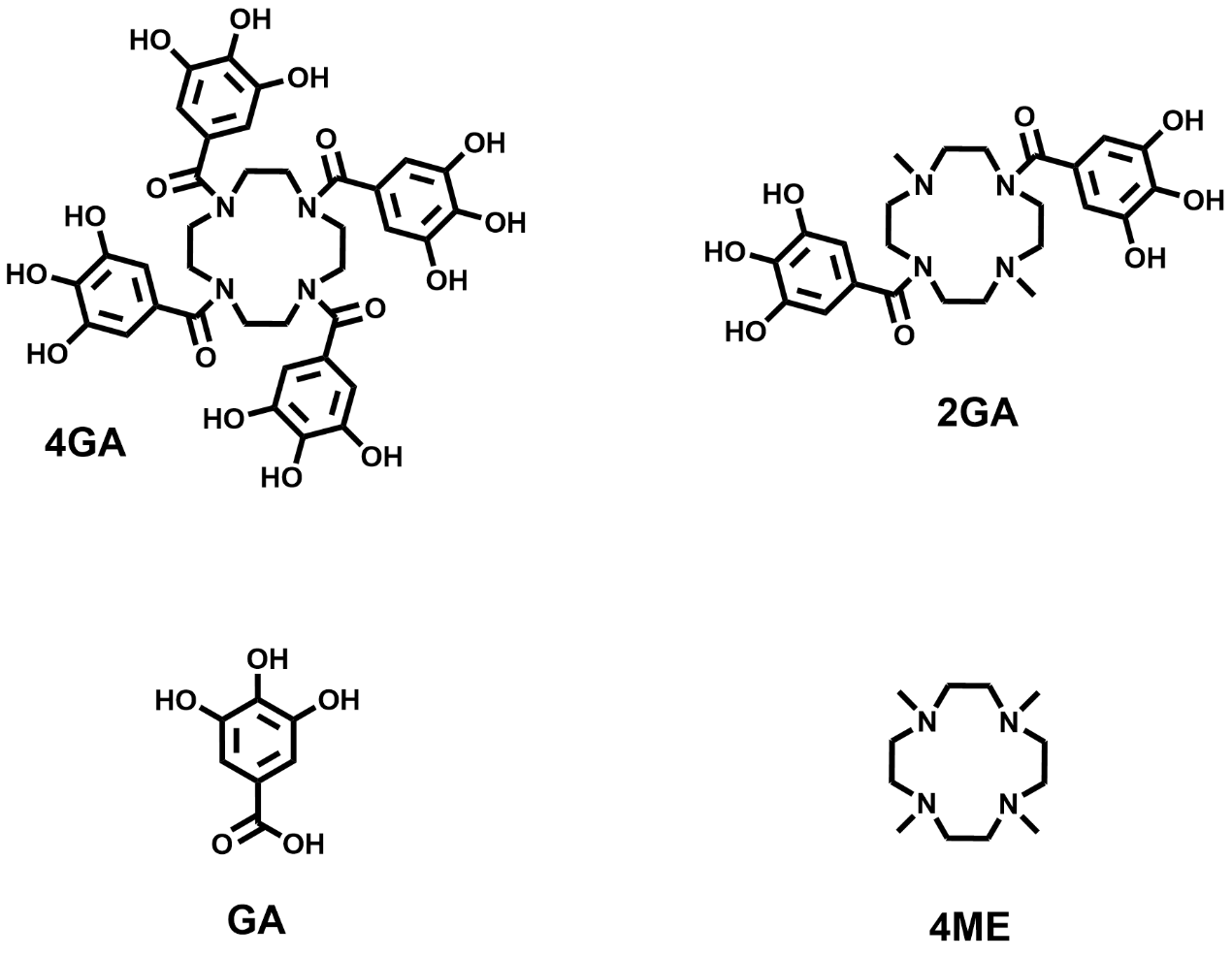


**Fig. S1** The compound structure of 4GA/2GA/GA/4ME.

2.Spectrastabilities of the new molecules


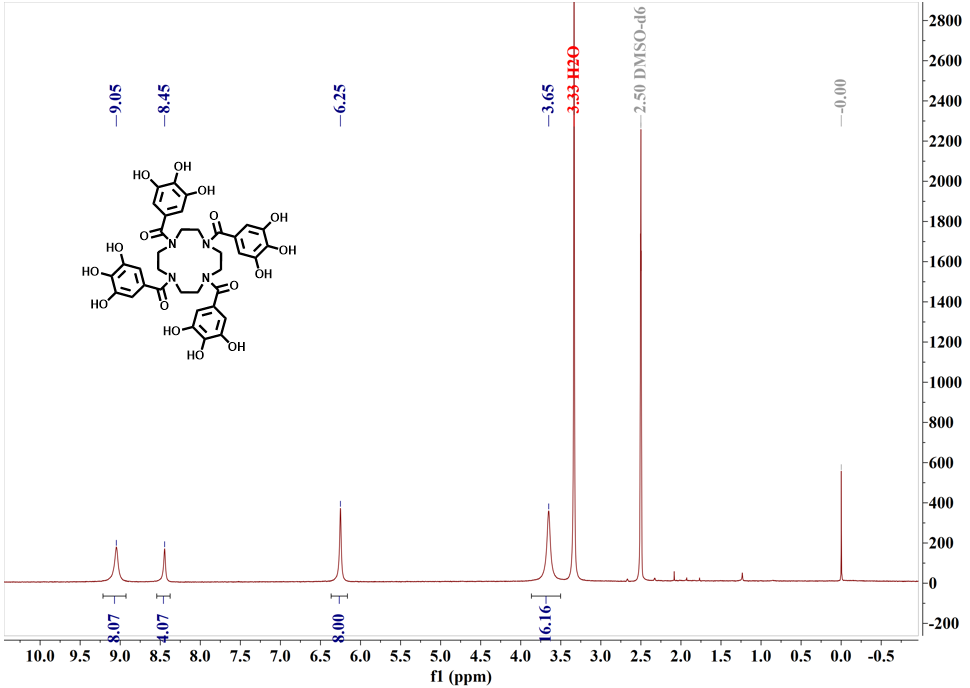


**Figure S2.** ^1^H NMR spectra of **4GA** (400 MHz, DMSO-*d_6_*).


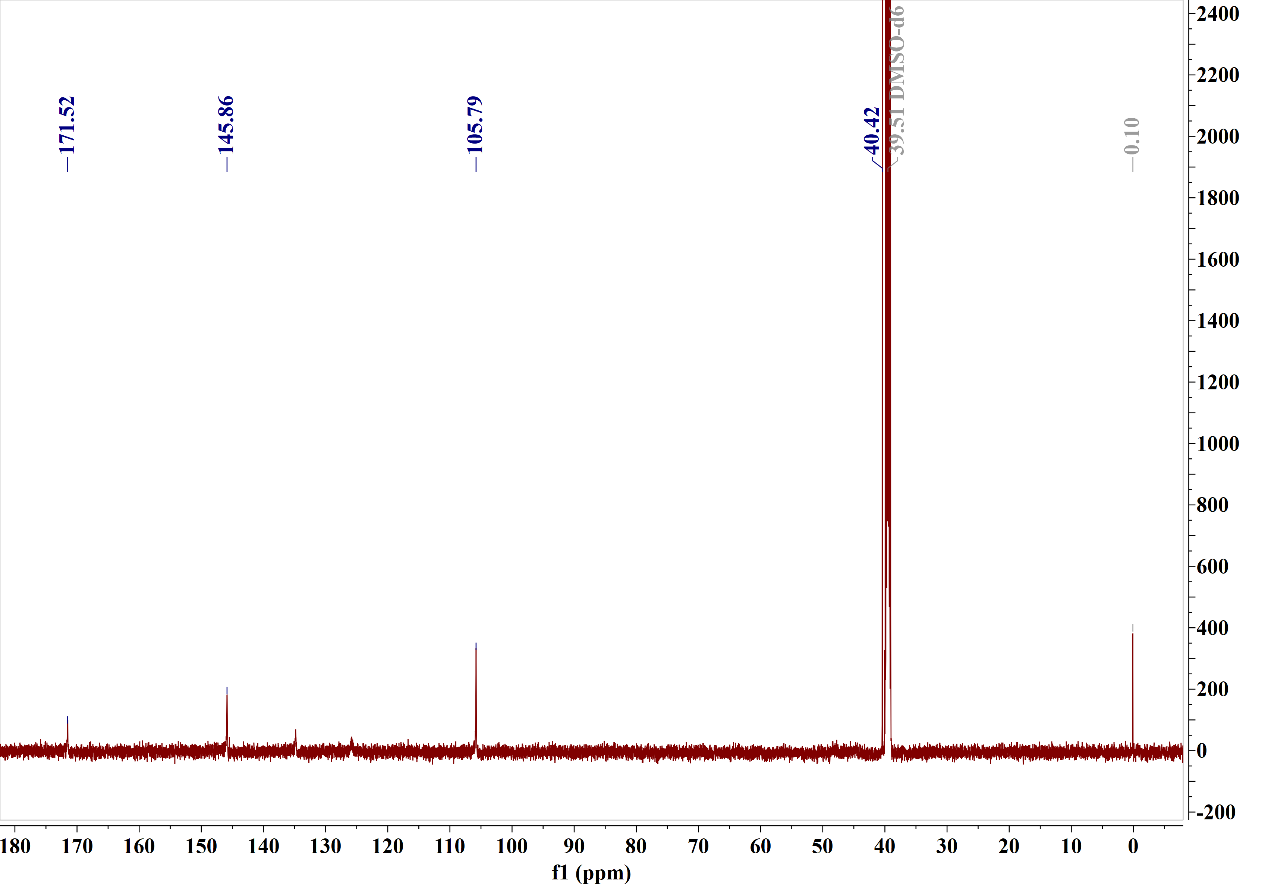


**Figure S3.** ^13^C NMR spectra of **4GA** (400 MHz, DMSO-*d_6_*).


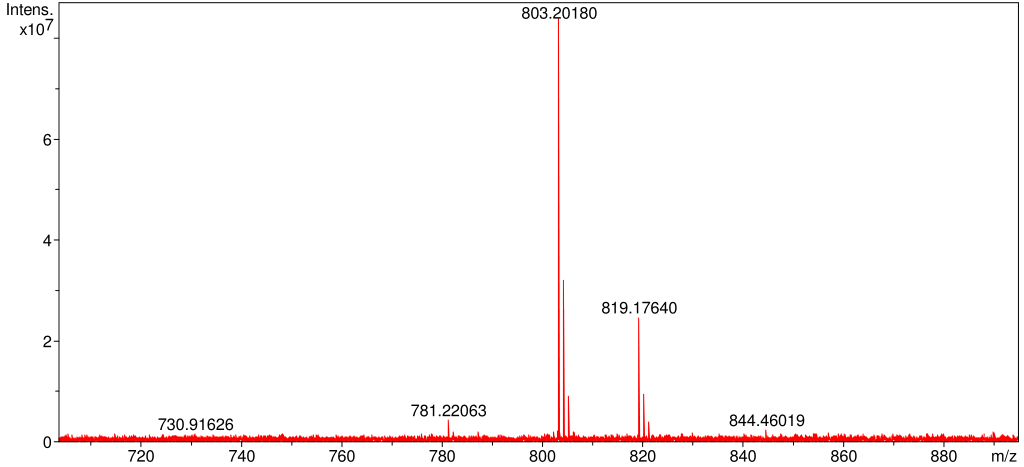


**Figure S4.** High resolution mass spectrum (HRMS) of **4GA** (CH_3_OH, by JEOL-JMS-T100LP AccuTOF).


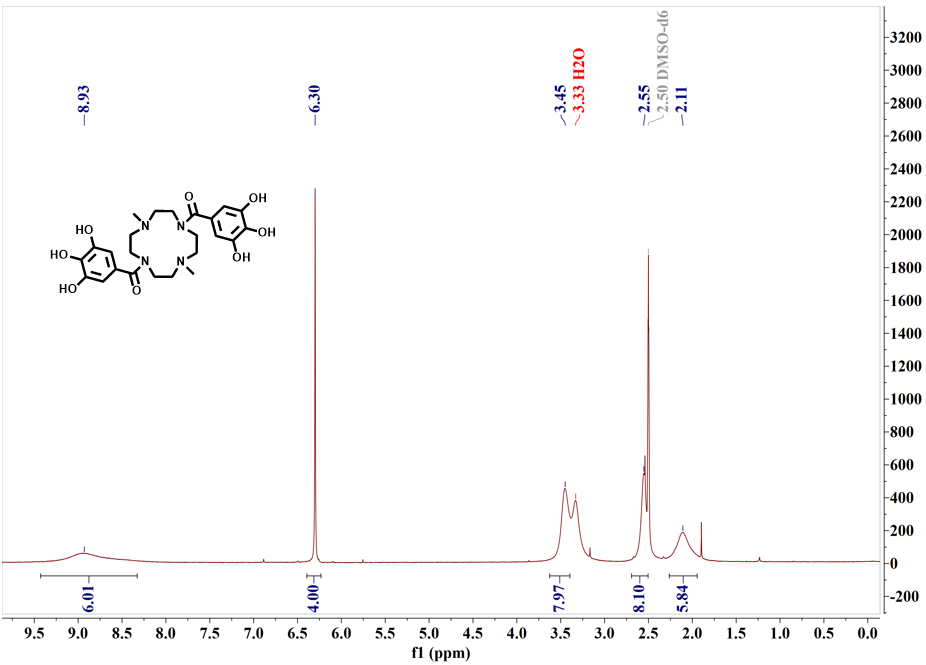


**Figure S5.** ^1^H NMR spectra of **2GA** (400 MHz, DMSO-*d_6_*).


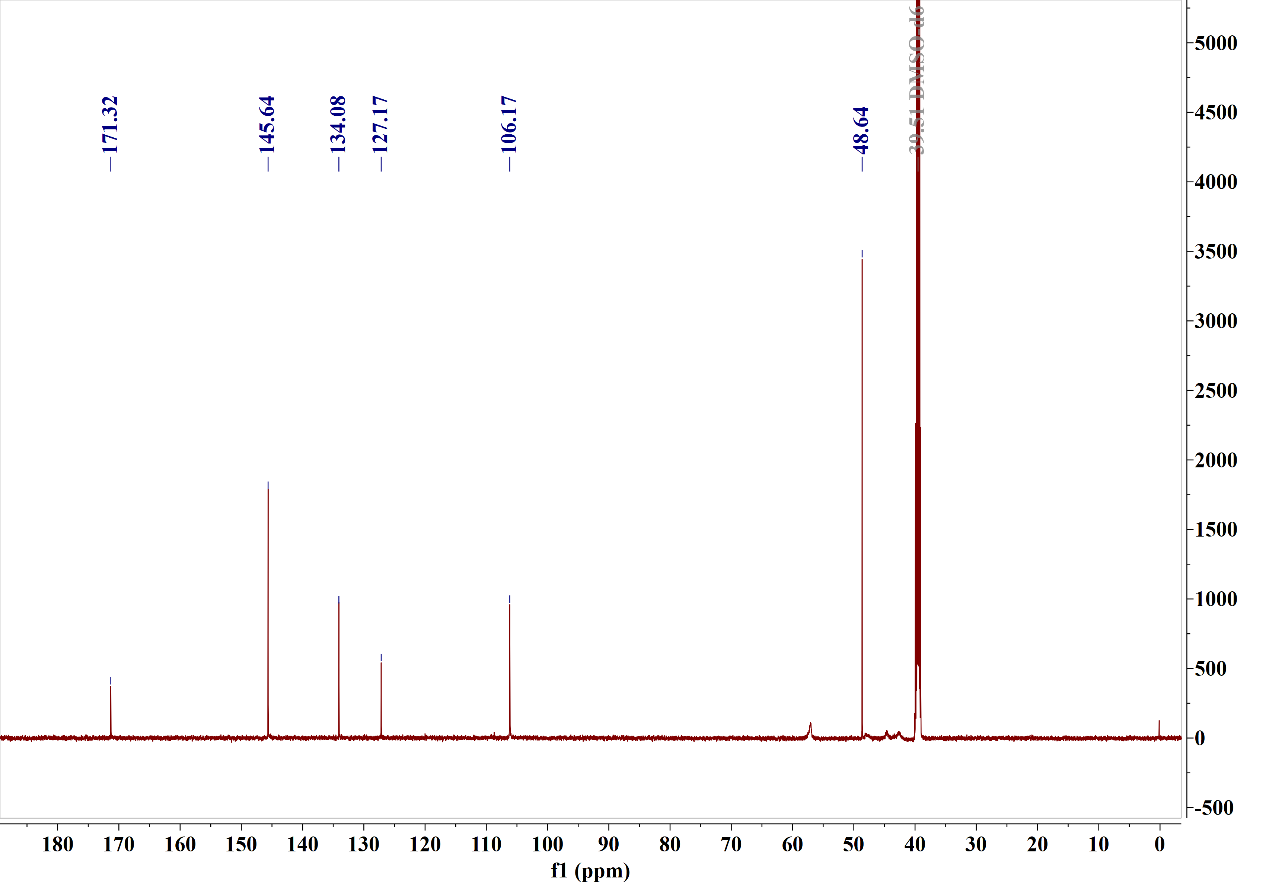


**Figure S6.** ^13^C NMR spectra of **2GA** (400 MHz, DMSO-*d_6_*).


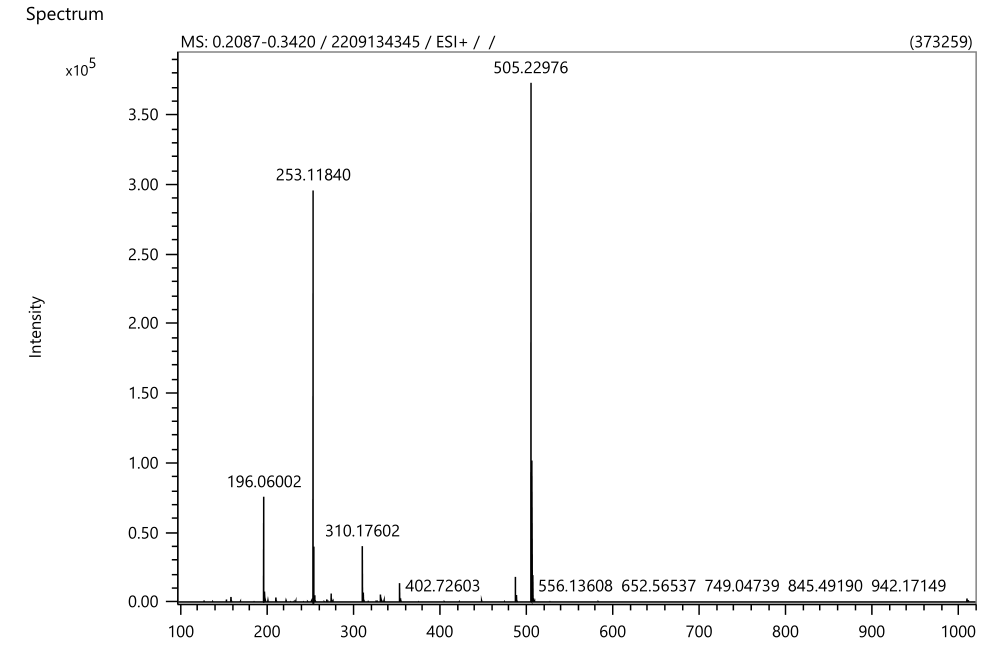


**Figure S7.** High resolution mass spectrum (HRMS) of **2GA** (CH_3_OH, by JEOL-JMS-T100LP AccuTOF).

3. Stabilities of the new molecules


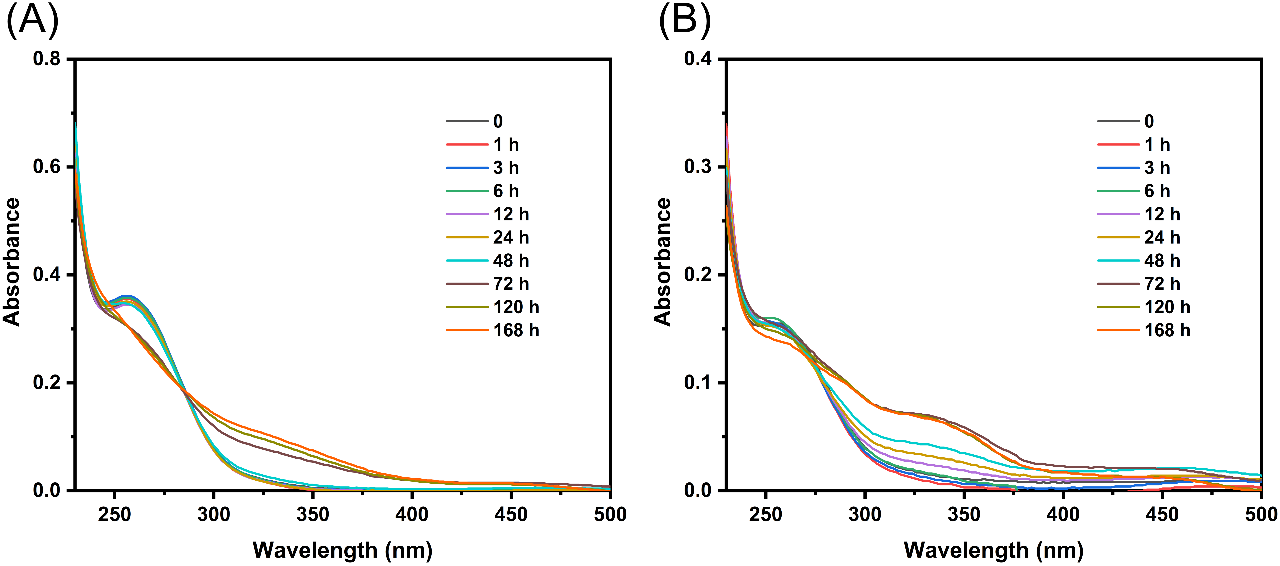


**Figure S8.** The stability of 4GA (A) and 2GA (B) in HEPES (50 mM, pH 7.4, 0.2% DMSO) verified by UV-vis absorption spectroscopy. All sample concentrations were 10 μM and kept at 37℃ in atmosphere.

3. Job’s plot analysis for determination of coordination stoichiometry of N-methylated cyclen (the precursor compounds) with Cu^2+^.

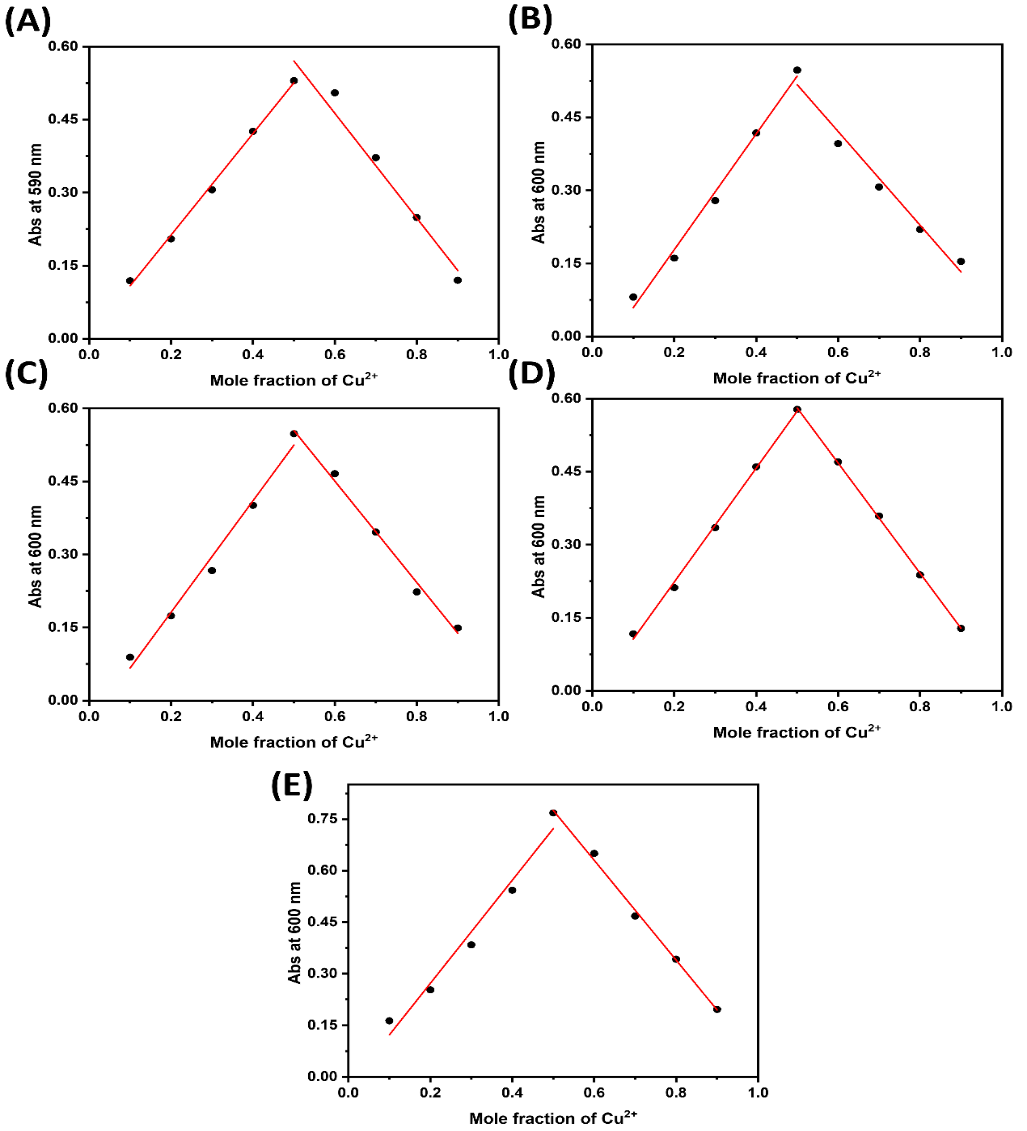


**Figure S9.** The chemical structure of cyclen derivates (upper), and the determination of the stoichiometry of complex (A) cyclen-Cu^2+^, (B) 1ME-Cu^2+^, (C) 2ME-Cu^2+^, (D) 3ME-Cu^2+^, (E) 4ME-Cu^2+^ (5 mM) by Job’s method.

4. Intrinsic Fluorescence quenching experiments for determination of tau protein quenching constants

The tyrosine fluorescence quenching experiment was carried out to determine the binding ability between inhibitors and R3 protein. If there exists combination between tyrosine residues on R3 protein and inhibitors, the change of tyrosine conformation will occur and the decline of fluorescence will be observed, indicating that the tyrosine residues might be one of the binding sites. Furthermore, this experiment is reasonable given the relatively weak fluorescence intensity of 4GA, 2GA, and GA (Figure S10). With titration of inhibitors into the solution of R3 protein, the fluorescence of tyrosine residues was gradually quenched in two temperatures of 25°C and 37°C, implying that **4GA** or **2GA** can interact with R3. Meanwhile, the tyrosine fluorescence spectra of GA were difficult to discover trends, demonstrating no significant interaction between GA and R3.

Fluorescence quenching usually proceeds through either dynamic quenching or static quenching. Dynamic quenching refers to the contact process between excited fluorophore and quencher, while static quenching refers to the formation of fluorophore-quencher complexes. A series of data processing about the fluorescence change of tyrosine residues at 25°C and 37°C was utilized to figure out the binding constant. Compared to the standard Stern-Volmer curves, a minor positive deviation from linearity was observed by plotting *F*_0_/*F* versus concentration, indicating a mixture of dynamic and static patterns (Figure S11). Furthermore, the Hill equation was used to calculate the quenching constant and binding sites of inhibitors on R3 protein (Figure S12). The analysis of the quenching data on the Hill equation showed that the quenching constant *K*_b_ both **4GA** and **2GA** was strongly promoted by increasing temperature, indicating that dynamic quenching plays a dominant role in this binding mode.





**Figure S10.** The fluorescence intensity of 4GA, 2GA, and GA (excitation, 275 nm).


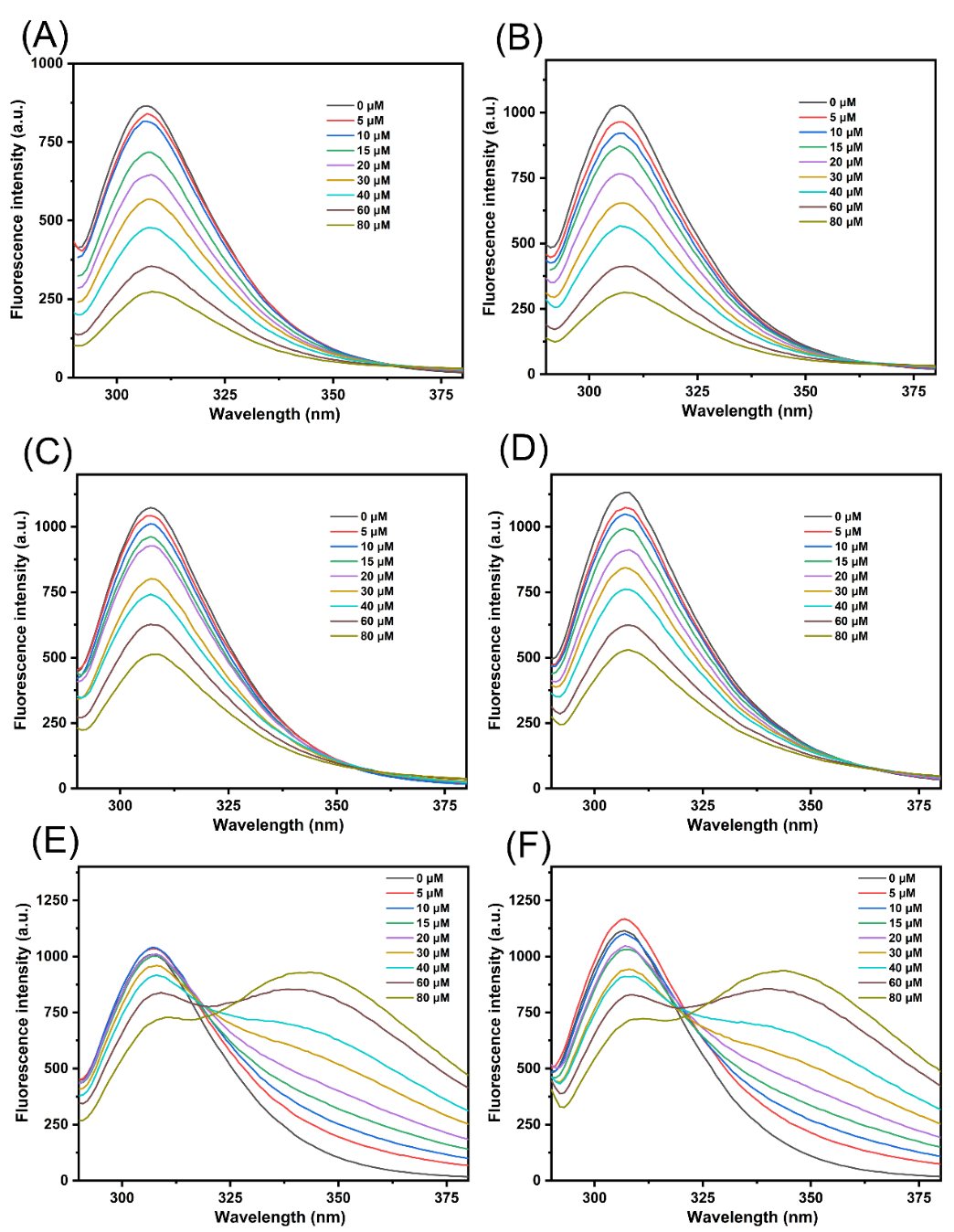


**Figure S11.** Intrinsic fluorescence quenching of R3 by different concentrations of (A) 4GA at 37℃, (B) 4GA at 25℃, (C) 2GA at 37℃, (D) 2GA at 25℃, (E) GA at 37℃, (F) GA at 25℃.


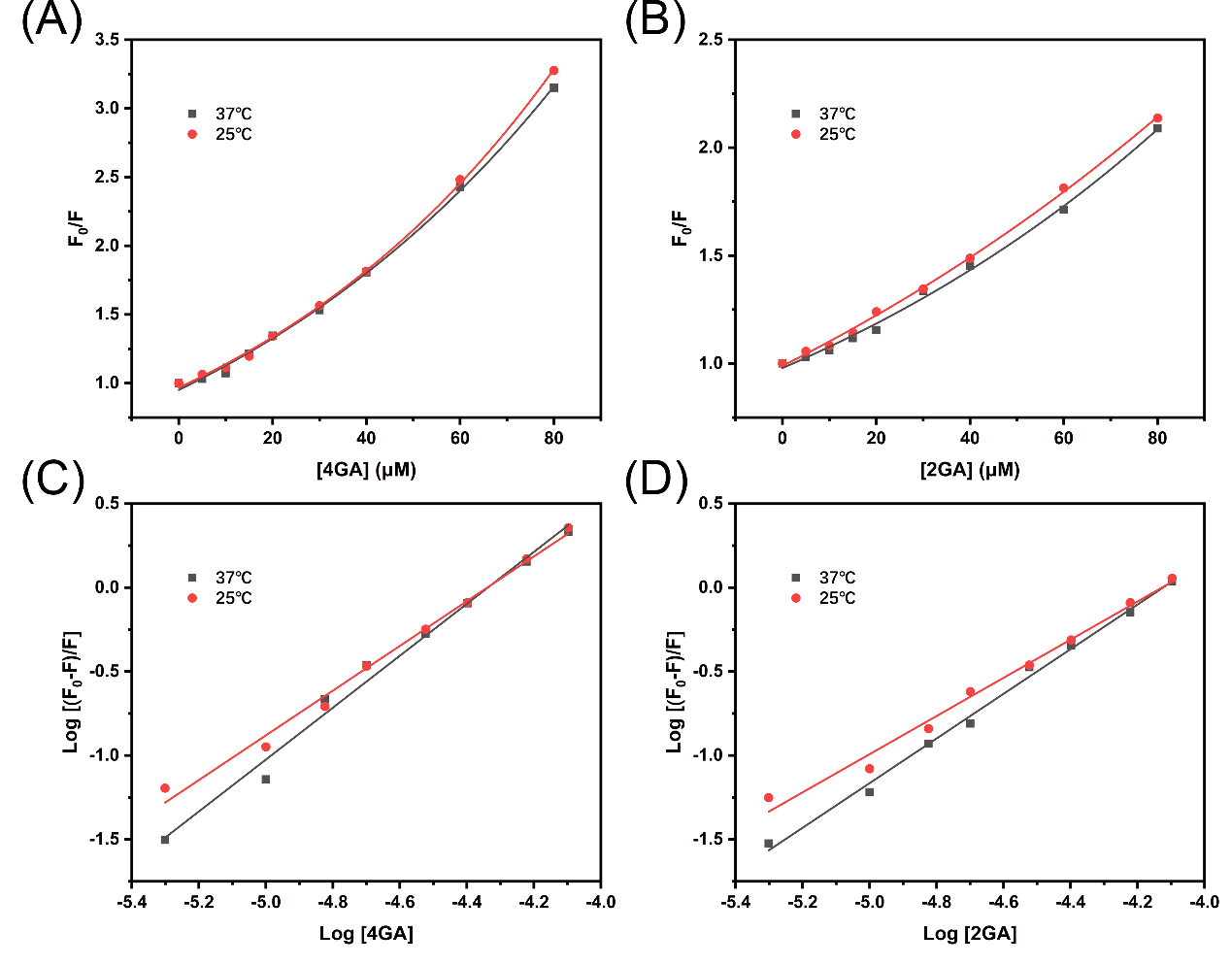


**Figure S12.**, Modified Stern-Volmer plot of R3 protein fluorescence quenching at different concentrations of (A) **4GA** and (B) **2GA**, respectively. Hill plot of R3 protein fluorescence quenching at different concentrations of (C) **4GA** and (D) **2GA**, respectively.

5. The dose−response inhibitory effects of different N-substituted azacrown on R3 aggregation investigated by ThS fluorescence. (For calculation of IC50)

Tau peptide R3 was adjusted to a concentration of 15 μM using Tris−HCl buffer (pH 7.4), and the synthetic compounds (**4GA**, **2GA,** together with 4ME, GA as the comparison) at different concentrations, as aggregation inhibitor, were added into the reaction mixture separately. Aggregation was induced by heparin, and incubated at 37 °C for 3 h. By introducing fluorescence probe ThS, the dose−response inhibitory effects on R3 aggregation were recorded, according to normalized ThS fluorescence intensity (excitation: 440 nm, emission: 500 nm).


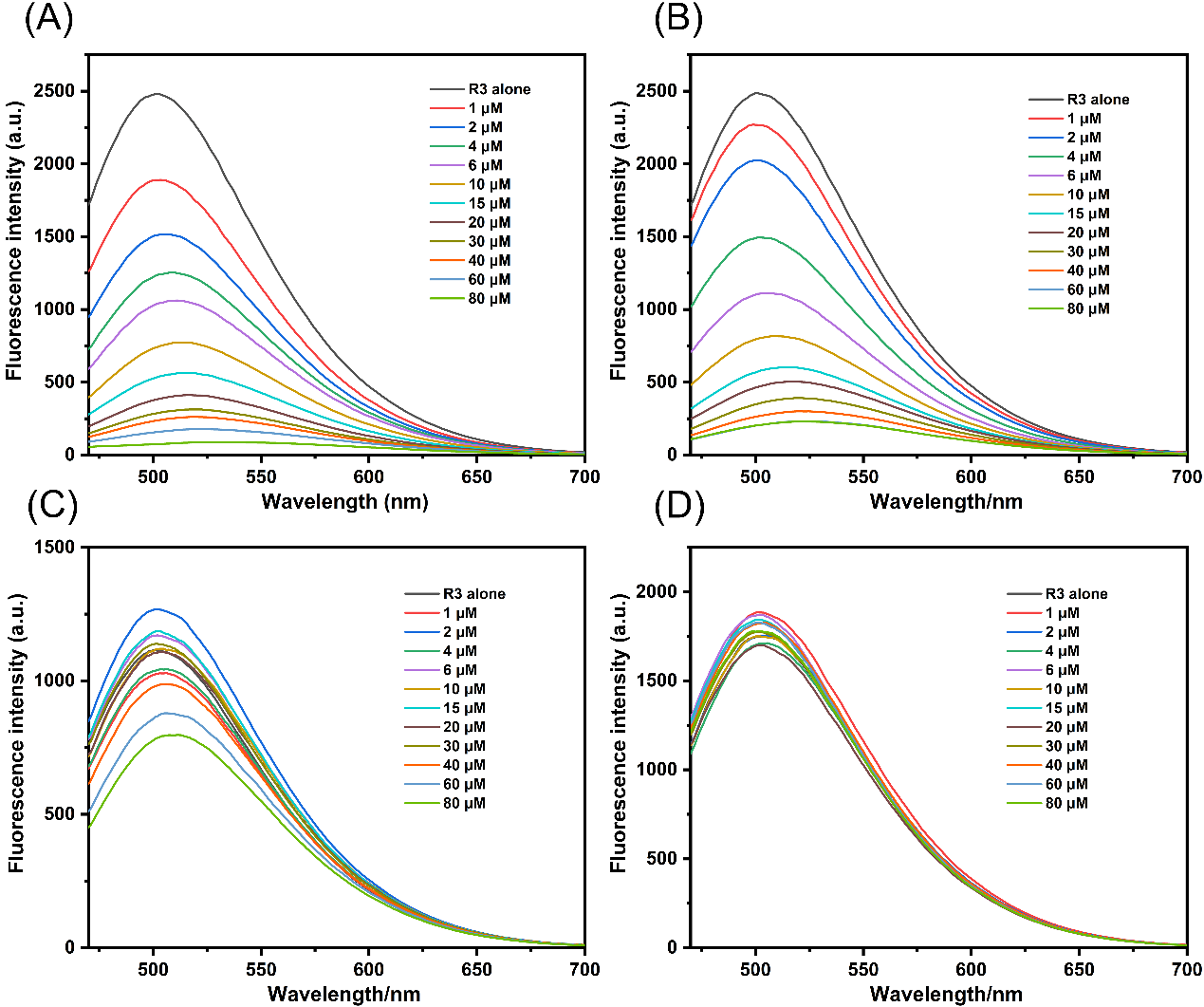


**Figure S13.** Spectra of ThS fluorescence for the R3 aggregation systems, and incubated with (A) 4GA, (B) 2GA, (C) GA, and (D) 4ME.

6. Inhibitory effects of cyclen and N-methylated cyclen on heparin-induced R3 aggregation, examined by ThS fluorescence

**Figure S14.** Inhibitory effects of N-substituted Azacrown on heparin-induced R3 aggregation, exermined by ThS fluorescence: 15 μM R3 and 3.8 μM heparin with cyclen, 1ME, 2ME, 3ME, and 4ME (80 μM) was incubated respectively at 37°C for 3h. 10 μM ThS was added to each of the solution, Spectra of ThS fluorescence were recorded at Ex440 nm.

7. N-substituted azacrown suppress the promotion of Cu^2+^ on heparin-induced R3 aggregation


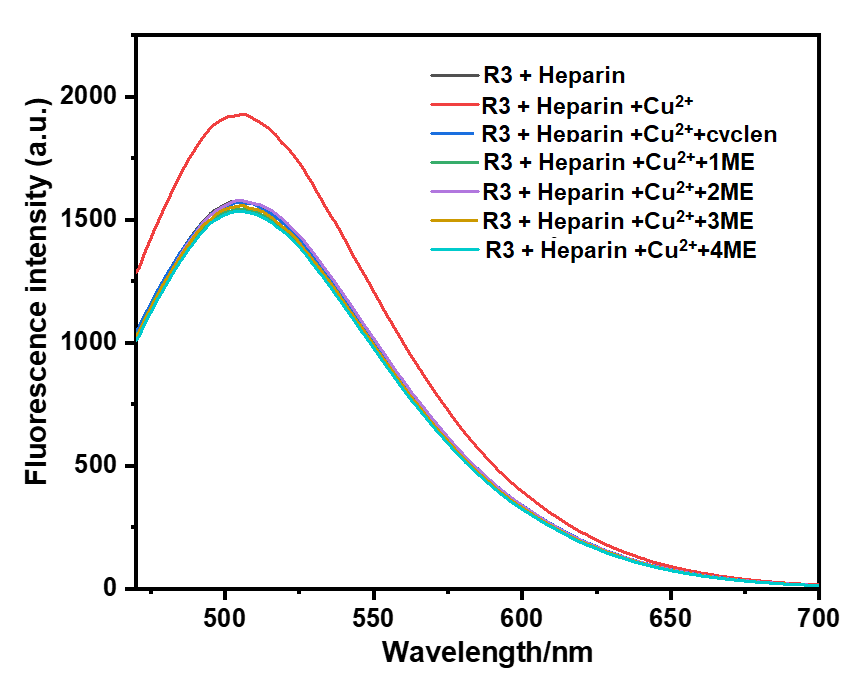


**Fig. S15** ThS fluorescence intensity of R3 (15 μM) incubated for 3 h. Final concentrations of R3, Cu^2+^, and compounds are 15, 3.8 and 10 μM, respectively. All aggregations were initiated by heparin (3.8 μM).

8. Quantitatively analysis of the fluorescence intensity in the Confocal laser scanning microscopy images in Figure 7 by ImageJ software





**Fig. S16** Quantitatively analysis of the fluorescence intensity in the Confocal laser scanning microscopy images (Figure 7) of SK-N-SH cells incubated with R3, heparin and different inhibitors by ImageJ software. Florescence intensity per cell (n = 8), analyzed by ImageJ software.

9. References

(1) Rodríguez-Rodríguez, A.; Regueiro-Figueroa, M.; Esteban-Gómez, D.; Rodríguez-Blas, T.; Patinec, V.; Tripier, R.; Tircsó, G.; Carniato, F.; Botta, M.; Platas-Iglesias, C. Definition of the Labile Capping Bond Effect in Lanthanide Complexes. *Chemistry – A European Journal* **2017**, *23* (5), 1110–1117.

(2) Rodríguez-Rodríguez, A.; Esteban-Gómez, D.; de Blas, A.; Rodríguez-Blas, T.; Fekete, M.; Botta, M.; Tripier, R.; Platas-Iglesias, C. Lanthanide(III) Complexes with Ligands Derived from a Cyclen Framework Containing Pyridinecarboxylate Pendants. The Effect of Steric Hindrance on the Hydration Number. *Inorg. Chem.* **2012**, *51* (4), 2509–2521.

(3) Ohashi, M.; Konkol, M.; Del Rosal, I.; Poteau, R.; Maron, L.; Okuda, J. Rare-Earth Metal Alkyl and Hydride Complexes Stabilized by a Cyclen-Derived [NNNN] Macrocyclic Ancillary Ligand. *J. Am. Chem. Soc.* **2008**, *130* (22), 6920–6921.

(4) Coates, J. H.; Hadi, D. A.; Lincoln, S. F. The Preparation, Characterization and Solution Chemistry of Some Nickel (II) and Copper (II) Complexes of 1,4,7,10-Tetramethyl- 1,4,7,10-tetraazacyclododecane. *Aust. J. Chem.* **1982**, *35*, 903-9.

(5) Ye, J.; Abiman, P.; Crossley, A.; Jones, J. H.; Wildgoose, G. G.; Compton, R. G. Building Block Syntheses of Gallic Acid Monomers and Tris-( *O* -Gallyl)-Gallic Acid Dendrimers Chemically Attached to Graphite Powder: A Comparative Study of Their Uptake of Al(III) Ions. *Langmuir* **2010**, *26* (3), 1776–1785.

(6) Tabassum, S.; Al-Asbahy, W. M.; Afzal, Mohd.; Arjmand, F. Synthesis, Characterization and Interaction Studies of Copper Based Drug with Human Serum Albumin (HSA): Spectroscopic and Molecular Docking Investigations. *J. Photochem. Photobiol. B, Biol.* **2012**, *114*, 132–139.

(7) Paul, B. K.; Ray, D.; Guchhait, N. Spectral Deciphering of the Interaction between an Intramolecular Hydrogen Bonded ESIPT Drug, 3,5-Dichlorosalicylic Acid, and a Model Transport Protein. *Phys. Chem. Chem. Phys.* **2012**, *14* (25), 8892.
